# Melatonin-related dysfunction in chronic restraint stress triggers sleep disorders in mice

**DOI:** 10.3389/fphar.2023.1210393

**Published:** 2023-06-20

**Authors:** Tian-Ji Xia, Zhi Wang, Su-Wei Jin, Xin-Min Liu, Yong-Guang Liu, Shan-Shan Zhang, Rui-Le Pan, Ning Jiang, Yong-Hong Liao, Ming-Zhu Yan, Li-Da Du, Qi Chang

**Affiliations:** ^1^ Institute of Medicinal Plant Development, Chinese Academy of Medical Sciences and Peking Union Medical College, Beijing, China; ^2^ Institute of Drug Discovery Technology, Ningbo University, Ningbo, China; ^3^ Institute of Molecular Medicine and Innovative Pharmaceutics, Qingdao University, Qingdao, China; ^4^ Department of Surgery, University of Toronto, Toronto, TO, Canada

**Keywords:** chronic restraint stress, sleep disorders, melatonin, sleep fragmentation, circadian rhythm, insomnia

## Abstract

Stress may trigger sleep disorders and are also risk factors for depression. The study explored the melatonin-related mechanisms of stress-associated sleep disorders on a mouse model of chronic stress by exploring the alteration in sleep architecture, melatonin, and related small molecule levels, transcription and expression of melatonin-related genes as well as proteins. Mice undergoing chronic restraint stress modeling for 28 days showed body weight loss and reduced locomotor activity. Sleep fragmentation, circadian rhythm disorders, and insomnia exhibited in CRS-treated mice formed sleep disorders. Tryptophan and 5-hydroxytryptamine levels were increased in the hypothalamus, while melatonin level was decreased. The transcription and expression of melatonin receptors were reduced, and circadian rhythm related genes were altered. Expression of downstream effectors to melatonin receptors was also affected. These results identified sleep disorders in a mice model of chronic stress. The alteration of melatonin-related pathways was shown to trigger sleep disorders.

## 1 Introduction

Sleep affects various physiological homeostasis and has a pivotal role in regulating the central nervous system functions (Tononi and Cirelli, 2014; Cuddapah et al., 2019). Sleep disorders are commonly associated with neurological pathogenesis and may indicate some neurodegenerative processes (Musiek and Holtzman, 2016). Sleep disorders include insomnia, narcolepsy, sleep apnea syndrome, circadian rhythm disorders, etc., affecting up to 40.49% population during 2019–2021 and becoming a significant public health concern (Jahrami et al., 2022). Insomnia, the most prevalent type of sleep disorder, is characterized by difficulty in sleep initiation and maintenance (Riemann et al., 2020). Circadian rhythm disorders include advanced or delayed sleep phase disorder and irregular sleep-wake rhythm (Allega et al., 2018). Sleep disorders are tightly linked with functional consequences like decreased attention and concentration, an increased risk of disease, lower work productivity, etc (Battle, 2013). Sleep is sensitive and can easily be affected. Predisposing factors for sleep disorders include age, sex, environment, stress, irregular sleep-wake schedule, etc (Billings et al., 2020; Meyer et al., 2022). A study has indicated that poorer sleep is associated with elevated feelings of stress in both individuals with sleep disorders and individuals from the general population (Demichelis et al., 2022). Increased levels of stress at night lead to decreases in slow wave sleep, sleep efficiency, and general sleep quality (Kim and Dimsdale, 2007). Stressful life events, related to work/school living, family, health, or indeterminate with negative emotional valence, are the most frequent triggers of insomnia (Bastien et al., 2004; Drake et al., 2014). In addition, many neuropsychiatric disorders, especially depression, can lead to sleep disorders. Sleep disorders are the most prominent symptom in depressive patients (Fang et al., 2019).

Sleep is regulated by circadian and homeostatic processes (Saper et al., 2005; Borbély, 2022). Melatonin (N-acetyl-5-methoxytryptamine) is an endogenous hormone that interacts with membrane melatonin receptors MT1 and MT2, two different types of G-protein-coupled receptors expressed both in brain regions and peripheral tissues (Pandi-Perumal et al., 2008; Pohanka, 2022). Melatonin regulates the circadian rhythm and affects sleep architecture. The central actions of melatonin on circadian rhythms focus on the hypothalamus, especially the suprachiasmatic nucleus (SCN) (Slominski et al., 2012). Melatonin also functions on the cortex, cerebellum, hippocampus, substantia nigra, and ventral tegument. Melatonin provides feedback signals to the SCN, having two distinct actions on the suprachiasmatic circadian clock, an acute inhibitory effect on neuronal firing and a phase-shifting effect on rhythm to regulate sleep (Liu et al., 1997; Cruz-Sanabria et al., 2023). The SCN neurons exhibit spontaneous circadian rhythms by interacting with positive and negative transcriptional-translational feedback loops (TTFLs), which contain clock genes and proteins. The positive transcriptional part of TTFLs consists of brain and muscle ARNT-like 1 (Bmal1) and circadian locomotor output kaput (Clock). Bmal1/Clock heterodimers bind to E-box components of the promoters of the Period (Per1-3), Cryptochrome (Cry1, 2), and Reverb (Nr1d1, Nr1d2) genes, whose proteins suppress the positive part of the loop (Musiek and Holtzman, 2016). At the molecular level, melatonin affects the circadian expression of clock genes. The mRNA transcription patterns of Per1 and Bmal1 in the rat SCN are phase shifted during the second night after 1 mg/kg melatonin injection (Poirel et al., 2003).

The function of melatonin is related to its central and peripheral concentrations. It has been proved that exogenous melatonin administration during the daytime increases subjective sleepiness (Lok et al., 2019). Melatonin synthesis and secretion occur in the pineal gland (Klein and Moore, 1979). The synthesis of melatonin involves several enzymes and small molecular compounds. Firstly, tryptophan (Trp) is transported into the cell and forms serotonin (5-hydroxytryptamine, 5-HT) by tryptophan-5-hydroxylase and 5-hydroxytryptophan decarboxylase. Then, 5-HT is acetylated by aralkylamine-N-acetyltransferase and methylated by N-acetylserotonin-O-methyltransferase to form melatonin (Acuña-Castroviejo et al., 2014).

It has been widely agreed that sleep disorders are linked to stress (Åkerstedt et al., 2012; Veeramachaneni et al., 2019). In clinical observations, chronic stress is one of the key risk factors for depression (Cruz-Pereira et al., 2020). Sleep disorder is a symptom of depression (Steiger and Pawlowski, 2019). The potential mechanisms between depression and sleep disorders have been associated with several pathways, including inflammation, neurotransmitters, and circadian rhythm, but the precise molecular mechanism of how depression induces sleep disorders remained unclear (Fang et al., 2019). Therefore, to elucidate the specific mechanism underlying the sleep disorders caused by chronic stress, we used a chronic restraint stress (CRS) mouse model to simulate chronic daily stress in humans.

Numerous studies have shown that CRS treatment can cause physiological and psychological stress in rodents by restricting movement in narrow spaces (Campos et al., 2013). CRS is a commonly used model of depression, and 28 days of such treatment causes depression in animals (Lin et al., 2021; Zhou et al., 2021). Some articles have studied the effects of short-term (<2 weeks) restraint stress on sleep in mice, but no long-term studies. (Meerlo et al., 2001; Koehl et al., 2006; Tang et al., 2007; Xu et al., 2023). In the present study, we built a mouse model by CRS 10 h each day for 28 days mimicking chronic stress, long-term stress-associated sleep structure, and disrupted circadian rhythm in patients. We then focused on melatonin, a key sleep regulator, including its content, synthesis, interaction with receptors, and regulation of circadian rhythm genes and proteins. Our study aims to provide a route for developing better treatment for sleep disorders.

## 2 Materials and methods

### 2.1 Animals modeling

Male ICR mice (4-week-old, weighing 20–22 g) were purchased from Beijing Vital River Laboratory Animal Technology Co., Ltd. (Beijing, China) and randomly assigned into different groups by weight to equalize the average weight in each group. Four mice were housed per cage, maintained at a constant temperature (25°C ± 0.5°C) and humidity (50% ± 10%) under an automatically controlled 12 h light/dark cycle, with *ad libitum* access to food and water. The light started at 9:00, defined as zeitgeber time (ZT) 0. All animal procedures were approved by the Animal Ethics Committee of the Institute of Medicinal Plant Development, Chinese Academy of Medical Sciences (approved No. SLXD-20210826023) and maintained adherence to the National Institutes of Health (NIH) guidelines for the care and use of laboratory animals.

**FIGURE 1:**
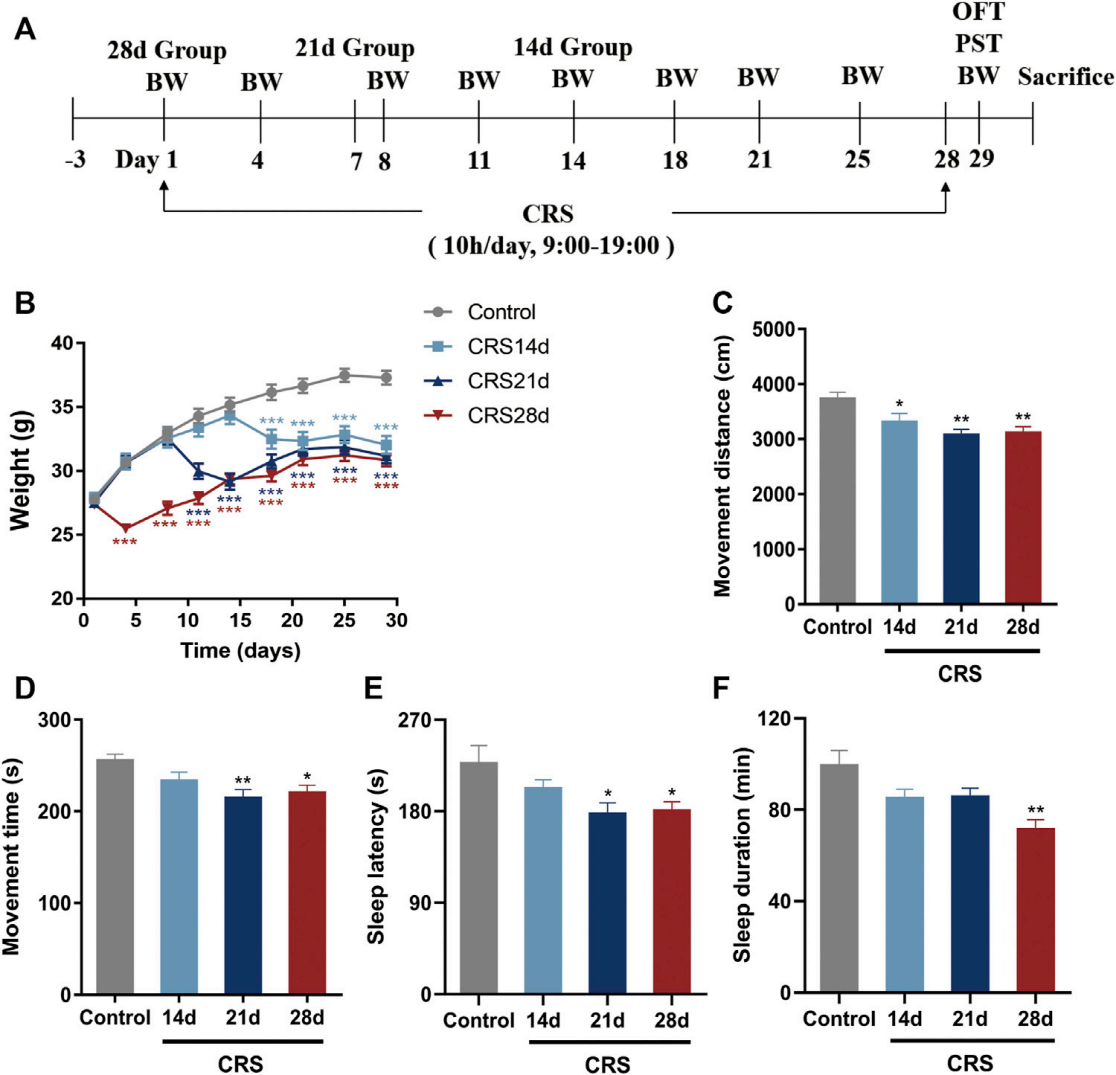
Effects of CRS on weight, locomotor activity, and sleep in mice (n = 10–12). **(A)** Experimental design of CRS-induced sleep disorders. Group CRS (28 days) was restrained on day 1 while group CRS (21 days) and group CRS (14 days) were restrained on day 8 and 15, respectively. **(B)** Body weight. **(C)** Movement distance of OFT. **(D)** Movement time of OFT. **(E)** Sleep latency. **(F)** Sleep duration. Data are presented as mean ± SEM. Compared with control group, *p < 0.05, **p < 0.01, ***p < 0.001.

After 3 days of acclimatization, the mice in the CRS-treated group were individually placed into a plastic cylinder (width: 3 cm) with holes at the bottom to allow free breathing (Wang et al., 2022), for 10 h (9:00–19:00) per day, for 14, 21, or 28 consecutive days (Figure 1A). The cylinder length was adjusted according to the mouse size to ensure complete restraint. The control mice did not receive food and water during the time the CRS-treated groups were subjected to stress. After CRS completion, the mice were returned to their cages with free access to food and water. The body weight (BW) of the animals was recorded every 3–4 days (Figure 1A).

### 2.2 Open field test

The open field test (OFT) was conducted on day 29 after modeling. The mice were placed individually in the center of Plexiglas boxes (30 × 28 × 35 cm) to record locomotor behaviors within 10 min after adapting to the new environment for 3 min. Locomotor activity was expressed as movement time (s) and distance (cm) using a video-tracking and analysis system (KSYY-OP-V4.0, Beijing, China).

### 2.3 Pentobarbital-induced sleep test

After the OFT, the pentobarbital-induced sleep test (PST) was performed between ZT 4.0–8.0 on day 29. The mice were moved to the testing room to adapt for 1 h, after which mice were injected intraperitoneally with 40 mg/kg (subthreshold dose) pentobarbital sodium to observe and record the number of mice falling asleep in each group within 30 min (Zhong et al., 2021). When the mice lost the righting reflex for about 1 min, they were considered asleep. Other mice were treated with pentobarbital (65 mg/kg, i. p.) to measure the sleep latency (time between pentobarbital injection and sleep onset) and duration (time between righting reflex loss and recovery) (Dong et al., 2021). The observers scoring these variables were blinded to grouping.

### 2.4 Electrode implantation

Mice in the control (n = 6) and CRS 28 d (*n* = 6) groups were anesthetized with 70 mg/kg pentobarbital and placed in a stereotaxic frame (RWD Life science Co., Ltd, Shenzhen, China). For electroencephalogram (EEG) monitoring, three stainless steel recording screws were implanted epidurally over the right frontal cortex (ML + 1.0 mm, AP + 1.5 mm from lambda) and the right parietal cortex (ML + 2.0 mm, AP—3.0 mm from lambda), and the cerebellum (ML 0.0 mm, AP—2.0 mm from bregma) as a reference electrode. For electromyography (EMG) monitoring, two flexible stainless-steel wire electrodes were placed bilaterally into the neck muscles to record postural tone. All screws were fixed on the skull surface with a dental base acrylic resin by connecting to a mini-connector to provide insulation and structural stability. Mice were allowed to recover in their cage for ≥10–14 days before EEG/EMG recordings. CRS-treated animals started with restraint on the second day after surgery.

### 2.5 EEG/EMG recording and analysis

Mice in the control (*n* = 6) and CRS 28 d (*n* = 6) groups were recorded individually in each chamber for 24 h (starting at ZT 0 on day 29) after acclimation. EEG/EMG signals were filtered (band passes EEG 0.5–49 Hz, EMG 10–100 Hz) and digitized at a sampling rate of 512 Hz using Sirenia Acquisition (Pinnacle Technology, Michigan, United States). EEG and EMG signals were subjected to spectral analysis by fast Fourier transformation using Sirenia Sleep Pro (Pinnacle Technology, Michigan, United States). Vigilance states were classified as wake, NREMS (non-rapid eye movement sleep), and REMS (rapid eye movement sleep) for each 10 s epoch according to EEG patterns of delta power (0.5–4 Hz), theta power (4.0–8.0 Hz), and the integral of EMG signals as previously described (Yasugaki et al., 2019). For each epoch, we first determined the brain state using a threshold algorithm. The state of wake included states with high EMG power or low delta power without elevated EMG activity. A state was classified as NREM if the delta power was larger than the delta threshold with low EMG power. A state was assigned as REM if the delta power was lower than the delta threshold with high theta power and low EMG power. Then, we manually verified the automatic sorting to ensure that all states were assigned correctly. Every epoch contained several states (wake, NREMS, REMS), defined as the state that lasted the longest time. The standard for one count of any state was ≥2 consecutive epochs having the same state.

### 2.6 Transcriptomic analysis

Mice in the control (*n* = 4) and CRS 28 d (*n* = 4) groups were anesthetized and sacrificed parallelly during ZT 2.0–3.0 on day 29. Total RNA from the hypothalamus was by Trizol Reagent (Solarbio, Beijing, China) and RNA-Seq analysis was performed. RNA quality was measured using NanoDrop 2000and8000, Agilent 2100 Bioanalyzer, Agilent RNA 6000 Nano Kit, and RNA was sequenced using Illumina NovaSeq 6000 platform. The per kilobase of exon model per million mapped fragments of gene expression for each sample was calculated using HTSeq. To obtain and adjust the *p*-values between samples, a negative binomial distribution test and false discovery rate estimation were used. Differentially expressed genes (DEGs) were defined as those having adjusted *p*-values <0.05.

### 2.7 Real-time quantitative PCR

Mice in the control (*n* = 6) and CRS 28 d (*n* = 6) groups were anesthetized and sacrificed in parallel during ZT 2.0–5.0 on day 29. Total RNA was extracted from the hypothalamus using Trizol Reagent (CW0580A; CWBIO, Beijing, China) according to the manufacturer’s protocol and cDNA was generated by reverse transcription using the All-in-One First-Stand cDNA Synthesis Kit (P31012, TransGen Biotech, Beijing, China) with 2 μg of RNA as template. Real-time quantitative PCR (qRT-PCR) was performed using the TransStart Top Green qPCR SuperMix Kit (O21225, TransGen Biotech, Beijing, China). Each sample was tested in triplicate, and the results of relative mRNA levels were calculated using the 2^−ΔΔCT^ method and normalized to the expression of the housekeeping gene *ß*-actin. qPCR primers were synthesized by Sango Biotech (Shanghai, China); sequences are shown in Table 1.

**TABLE 1:** Primer sequences for qRT-PCR.

Genes (mouse)	Forward primer (5′-3′)	Reverse primer (5′-3′)
*Mtnr1a*	ACC​GCA​ACA​AGA​AGC​TCA​GGA​AC	GAT​GTC​AGC​ACC​AAG​GGA​TAA​GGG
*Mtnr1b*	TCC​GCA​GGG​AGT​ACA​AGA​GG	CAC​CTT​CCT​TGA​CAG​GCA​CG
*Clock*	AGA​CGG​CGA​GAA​CTT​GGC​ATT​G	AAC​CTT​TCC​AGT​GCT​TCC​TTG​AGA​C
*Arntl*	GGA​CTT​CGC​CTC​TAC​CTG​TTC​AAA​G	TCG​TTG​TCT​GGC​TCA​TTG​TCT​TCG
*Per1*	CTC​CTC​CTC​CTA​CAC​TGC​CTC​TTC	TTG​CTG​ACG​ACG​GAT​CTT​TCT​TGG
*Per2*	CTG​CGG​ATG​CTC​GTG​GAA​TCT​TC	GGT​TGT​GCT​CTG​CCT​CTG​TCA​TC
*Per3*	AAA​GAT​CCT​GAC​CTC​GCC​CTA​CG	TTT​GTG​CTT​CTG​CCT​CTC​GCT​TC
*Cry1*	GCC​AGC​AGA​CAC​CAT​CAC​ATC​AG	CCA​GGG​AAG​GAA​CGC​CAT​ATT​TCT​C
*Cry2*	TGG​ACA​AGC​ACT​TGG​AAC​GGA​AG	GTA​GAA​GAG​GCG​GCA​GGA​GAG​G
*Nr1d1*	CGT​CAT​CCT​CTT​CAT​CCT​CCT​CCT​C	CTT​GGT​AAT​GTT​GCT​TGT​GCC​CTT​G
*Bhlhe40*	CAG​TAC​CTG​GCG​AAG​CAT​GAG​AAC	TCC​GAG​ACC​ACA​CGA​TGG​AGA​TG

### 2.8 Liquid chromatography-tandem mass spectrometry analysis

The levels of Trp and γ-aminobutyric acid (GABA) in the hypothalamus and cortex were measured by liquid chromatography-tandem mass spectrometry (LC-MS/MS) by using a Prominence LC system (SHIMADZU, Kyoto, Japan) connected with a QTRAP 5500 mass spectrometer (AB SCIEX, Foster City, CA). A Restek Ultra Aqueous C18 column (3 μm, 100 mm × 2.1 mm, Bellefonte, PA, United States) was used for chromatographic separation. Acetonitrile and water containing 0.1% formic acid constituted the mobile phase at an eluting rate of 0.4 mL/min. The hypothalamus and cortex samples were homogenized with 100 μL water and then the 50 μL homogenate was spiked with 20 μL 500 ng/mL acetaminol as internal standard and 10 μL trifluoroacetic acid to precipitate the protein. The mixture was blended and centrifuged at 4°C, 21,000 ×*g* for 20 min. Then, the supernatant was collected and a 2 μL aliquot was injected into the system.

### 2.9 Enzyme-linked immunosorbent assay (ELISA)

5-HT concentrations in the hypothalamus and cortex were detected using a Serotonin Assay Kit (H104-1-2, Nanjing Jiancheng Bioengineering Institute, Nanjing, China). The melatonin concentration in the hypothalamus and serum was measured with a Mouse Melatonin ELISA Kit (D721190, Sango Biotech, Shanghai, China). The process was carried out according to the manufacturer’s instructions.

### 2.10 Western blotting

Mice in the control (*n* = 6) and CRS 28 d (*n* = 6) groups were anesthetized and sacrificed in parallel during ZT 2.0–6.0 on day 29. Brain tissues were immediately removed on ice and frozen at −80°C until homogenization. First, the hypothalamus and cortex tissues were lysed in ice-cold RIPA buffer (Solarbio, Beijing, China) containing 1% protease and phosphatase inhibitor cocktail (CWBIO, Beijing, China) with a mechanical homogenizer (Sonics, Newton, United States). Samples were then centrifuged for 20 min at 4°C and 15,000 g. Total protein concentrations were measured using a BCA protein assay kit (Solarbio, Beijing, China) and normalized on the basis. An equivalent of 30–50 µg protein was subjected to 10% tris-glycine SDS-PAGE and transferred to nitrocellulose membranes (Pall Corporation, New York, United States). The membranes were blocked with 5% BSA or non-fat milk in TBST for 1 h at room temperature and incubated in specific primary antibodies at 4°C overnight. After washing with TBST three times, the membranes were incubated with HRP-labeled goat anti-mouse or anti-rabbit IgG (1:5000 dilution, CWBIO, Beijing, China) for 2 h at room temperature. Protein bands were visualized using a gel imaging system (Bio-Rad, Hercules, United States) with an enhanced ECL substrate (CWBIO, Beijing, China) and analyzed with ImageJ software.

Antibodies used were as follows: *ß*-actin (1:2000, AC006, ABclonal), Melatonin receptor 1A (1:100, SC-390328, Santa Cruz), Melatonin receptor 1B (1:500, ab203346, Abcam), PKCα (1:1,000, 2056T, Cell Signaling Technology), CREB (1:1,000, ab32515, Abcam), Phospho-CREB (Ser133) (1:1,000, 9198S, Cell Signaling Technology), CaMKII (1:1,000, #3362, Cell Signaling Technology), Phospho-CaMKII (1:200, sc-32289, Santa Cruz), Bmal1 (1:500, ET1705-5, HUABIO), Clock (1:500, T1704-82, HUABIO), Cry1 (1:1,000, ab104736, Abcam), Cry2 (1:500, 13997-1-AP, Proteintech), Per1 (1:500, HA500097, HUABIO), Per2 (1:1,000, 67513-1-Ig, Proteintech), and Per3 (1:500, HA500072, HUABIO).

### 2.11 Statistical analysis

All results are represented as the mean ± standard error of the mean (SEM) and analyzed using SPSS version 18.0 statistical software (SPSS, Inc., Chicago, United States). The normality and homogeneity were checked by Shapiro-Wilk test and Levene’s test. Differences in multiple groups were statistically analyzed with One-way ANOVA followed by least significant difference or Dunnett’s T3 test. The data in the two groups were compared using Student’s *t*-test. The Mann-Whitney test was used for data following a non-parametric distribution. Significance was expressed as *p < 0.05, **p < 0.01 or ***p < 0.001. Figures were created using GraphPad Prism version 7.0 software (San Diego, United States).

## 3 Results

### 3.1 Impacts of repeated CRS treatments on mice behavior

To explore the effect of CRS treatment on weight, locomotor activity, and sleep, as well as to determine the time required by CRS treatment to cause sleep disorders in mice, the mouse body weight was recorded every 3–4 days, and an OFT and pentobarbital-induced sleep test were conducted on day 29 (Figure 1A). Compared with mice in the control group, the weight of CRS-treated mice decreased significantly in the first 3–4 days of the restraint process (*p* < 0.001) (Figure 1B). The movement distance and time decreased significantly, especially in the mice treated with CRS for 21 and 28 days, indicating that the locomotor activity was impaired (Figures 1C,D). The falling asleep rate, sleep latency, and sleep duration in the CRS groups were decreased in comparison with the controls. All these indexes in the CRS 28 d group were significantly different from the control group (Table 2; Figures 1C–F). Particularly, the sleep duration decreased to 85.5%, 86.3%, and 71.9% that of control mice after CRS treatment for 14, 21, and 28 days, respectively. Thus, CRS treatment for 28 days induced sleep disorders in mice and was selected to study the underlying mechanism.

**TABLE 2:** The effect of CRS on the falling asleep rate of mice (*n* = 11–12).

Group	Number of groups	Number of mice falling asleep	Falling asleep rate (%)
Control	12	7	58.33
CRS 14d	12	9	75.00
CRS 21d	12	3	25.00
CRS 28d	11	2	18.18*

Compared with the control group, *p < 0.05.

### 3.2 CRS treatment caused sleep disorders in mice

**FIGURE 2:**
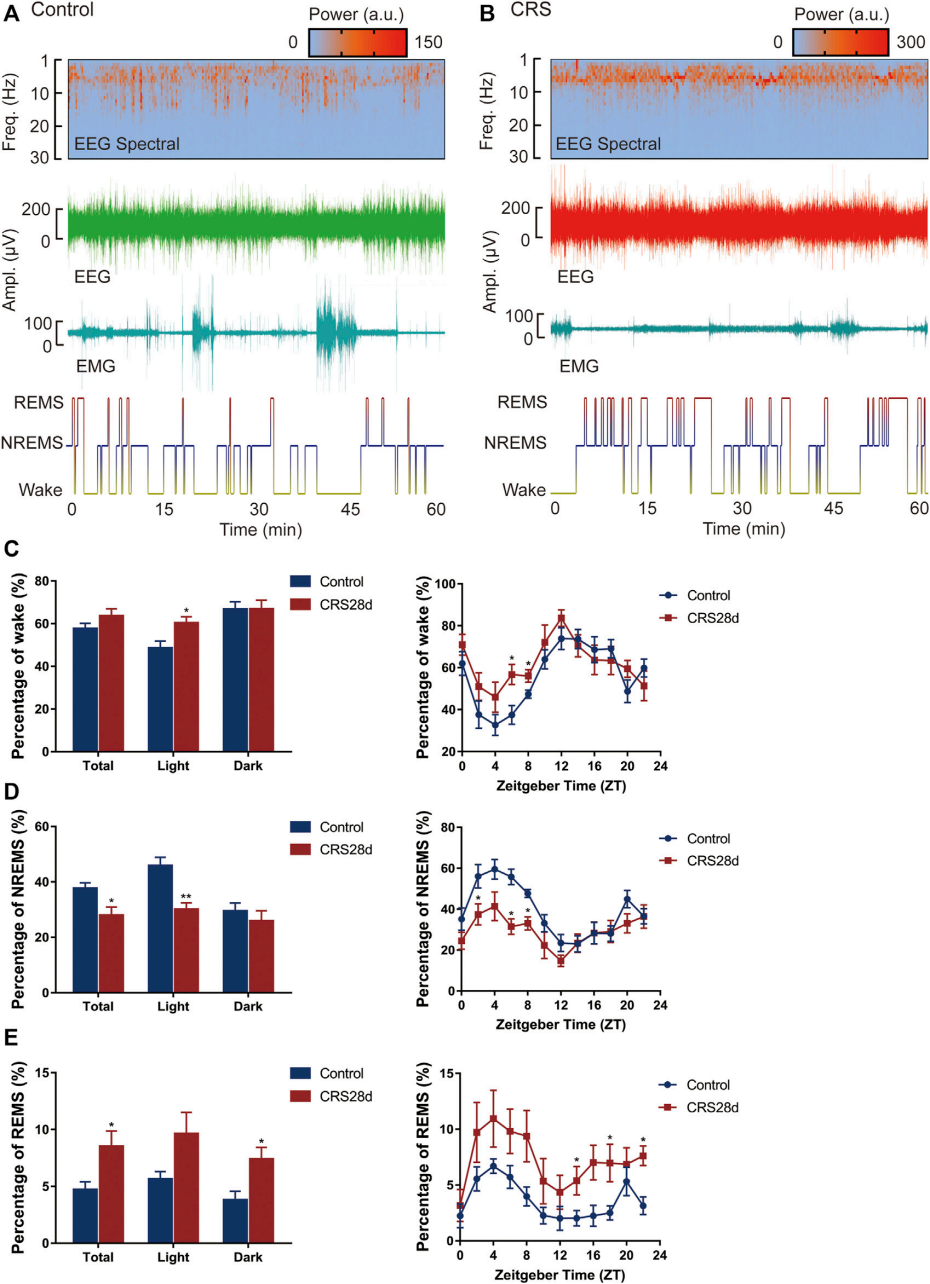
Effect of CRS 28 d on the sleep/wake cycle in mice (*n* = 6). **(A)** Example experiment. The EEG power spectrogram, EEG and EMG amplitude, and hypnogram of control mouse. **(B)** Example experiment. The EEG power spectrogram, EEG and EMG amplitude, and hypnogram of CRS mouse. **(C)** The percentage of wake amount. **(D)** The percentage of NREMS amount. **(E)** The percentage of REMS amount. Data were presented as mean ± SEM. Compared with control group, *p < 0.05.

The effect of CRS treatment on the sleep/wake architecture of the mice was investigated by consecutive EEG/EMG recordings for 24 h (started at ZT 0 on day 29). Brain states were classified from EEG and EMG recordings, including wake, REMS, and NREMS. REMS was observed at a high probability in CRS-treated mice. A tendency of increased wake frequency was also displayed in the CRS group (Figures 2A,B). Wake and NREMS are complementary in proportional changes in 24 h, the percentage of wakefulness was increased, especially during the light (Figure 2C). The percentage of NREMS was decreased significantly both in light and total. NREMS was mainly reduced in ZT 2–8, while wakefulness was significantly increased in ZT 6–8 (Figures 2C,D). In contrast to NREMS, the REMS percentage was increased significantly at ZT 14,18, and 22, mainly in the dark phase (Figure 2E). The alternations of percentage in each phase demonstrated disrupted circadian rhythm and insomnia in CRS-treated mice.

**FIGURE 3:**
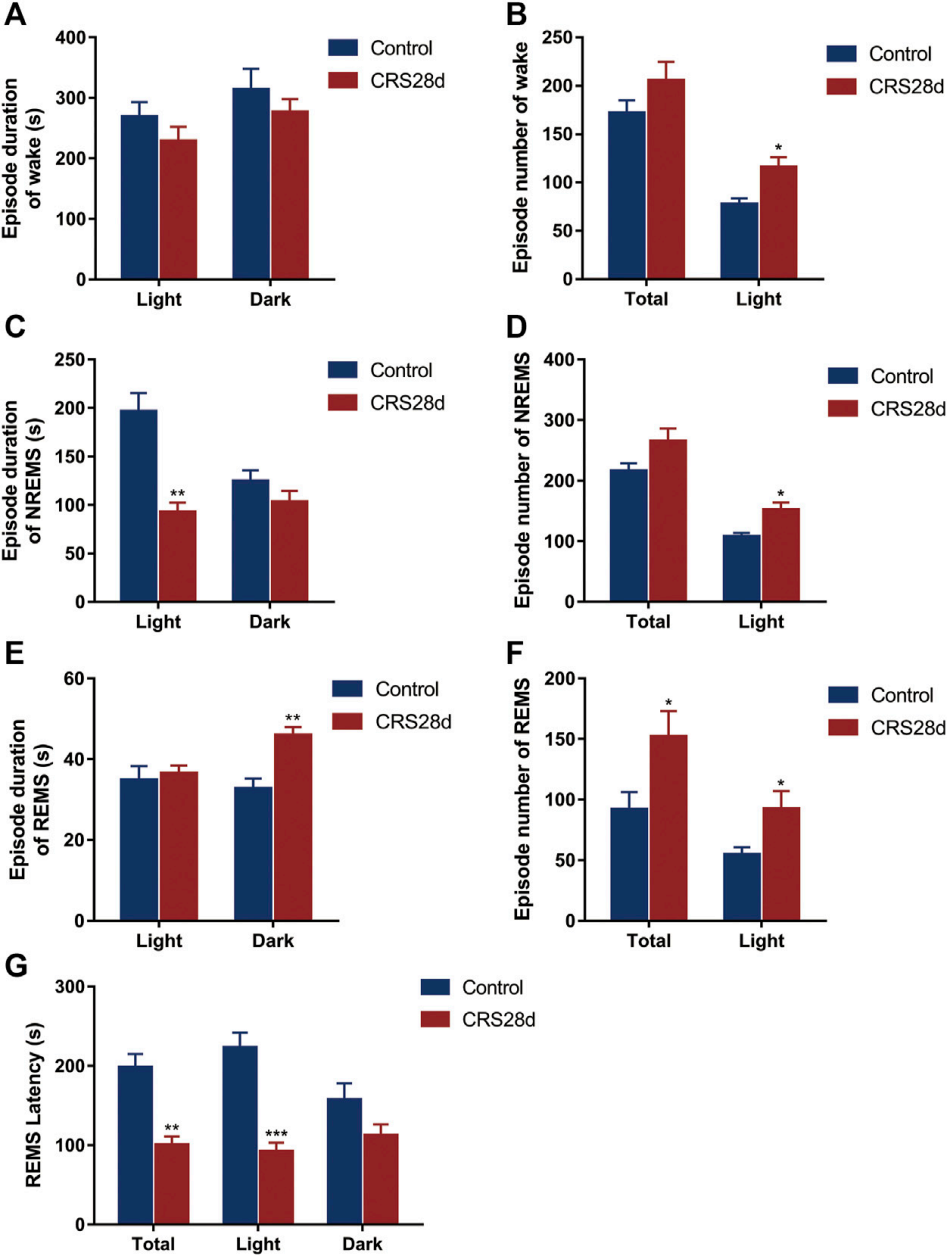
Effect of CRS 28 d on the episode duration and number of sleep/wake cycle in mice (*n* = 6). **(A)** Episode duration of wake time in light and dark phases. **(B)** Episode number of wake time in total and light phase. **(C)** Episode duration of NREMS time in light and dark phases. **(D)** Episode number of NREMS time in total and light phase. **(E)** Episode duration of REMS time in light and dark phases. **(F)** Episode number of REMS time in total and light phase. **(G)** Mean REMS latency in 24 h, light phase, and dark phase. Data were presented as mean ± SEM, *p < 0.05, **p < 0.01, ***p < 0.001.

Brain states and sleep structures are varied during light and dark phases (Figure 3). The mean duration of wake episodes was tended to decrease, and the episode numbers were increased significantly in the light phase (Figures 3A,B). Reversed episode duration changes were shown in NREMS and REMS episodes. The duration of NREMS was decreased significantly in the light phase, while REMS duration was increased significantly in the dark phase (Figures 3C,E). However, the number of NREMS and REMS episodes was increased significantly in light (Figures 3D,F). No alteration was found in the episode number of wake, NREMS, and REMS in dark. The increase in episode number in each phase explained that sleep in CRS-modeled mice was fragmented.

The REMS latency, i.e., duration of the NREMS episode immediately preceding a REMS episode, was examined from all groups. Accordingly, the REMS latency decreased not only over 24 h but also in the light phase (Figure G). Our EEG/EMG results indicated sleep fragmentation, circadian rhythm disorders, and insomnia in the CRS-treated mice.

### 3.3 Impacts of CRS treatment on hypothalamic gene expression profile

**FIGURE 4:**
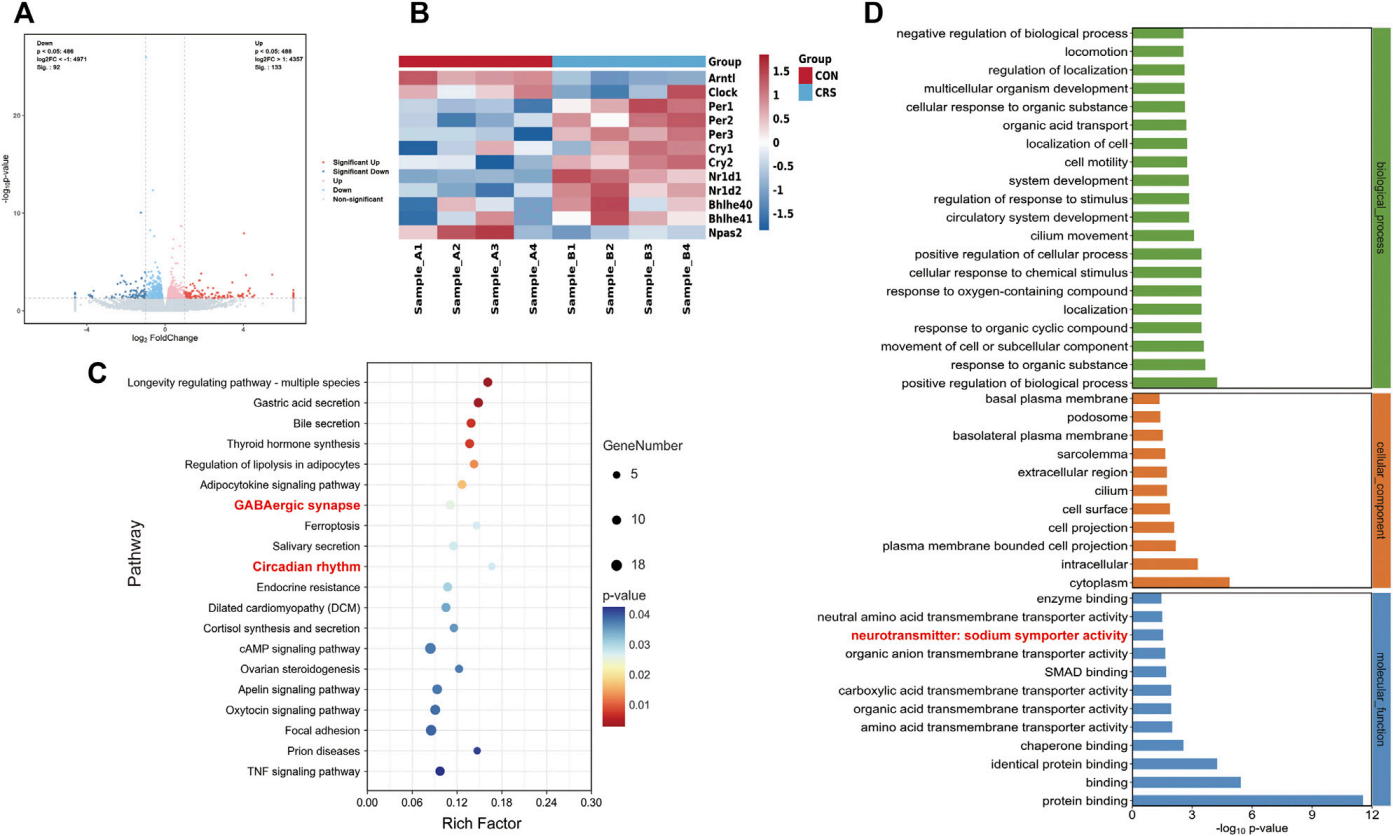
Effect of CRS 28 d on gene expression profile in the mouse hypothalamus (*n* = 4). **(A)** Volcano map of transcriptome. **(B)** Heatmap of the circadian rhythm signaling pathway-related DEG expression. **(C)** KEGG (Top 20 of the total pathway, *p* < 0.05). **(D)** Significantly enriched GO biological process, cellular component, and molecular function for DEGs.

Hypothalamic RNA sequencing was performed after 28 d-CRS treatment to gain insights into the molecular mechanisms whereby CRS treatment caused sleep disorders. We identified 225 DEGs between the control and CRS 28 d groups, including 92 genes significantly downregulated and 133 genes significantly upregulated (Figure 4A). Kyoto Encyclopedia of Genes and Genomes (KEGG) enrichment analysis showed the top 20 pathways of these DEGs (Figure 4C). In which, the pathways involved in sleep regulation were GABAergic synapse and circadian rhythm signaling pathways. The main alternation in GABAergic synapses was neurotransmitter symporter activity as shown in Figure 4D. GABA symporters were more relative to epilepsy and other neurological disorders than sleep. Therefore, we focused on circadian rhythm signaling pathways; the heatmap of circadian rhythm genes was highly regulated by CRS treatment (Figure 4B). GABA and circadian rhythms were both regulated by melatonin. Therefore, our transcriptome results indicated that melatonin was a key regulator in the sleep disorder caused by CRS treatment, and the GABA and melatonin levels were probably altered in the brain.

### 3.4 CRS treatment changed transcription and expression of hypothalamic circadian rhythm genes

**FIGURE 5:**
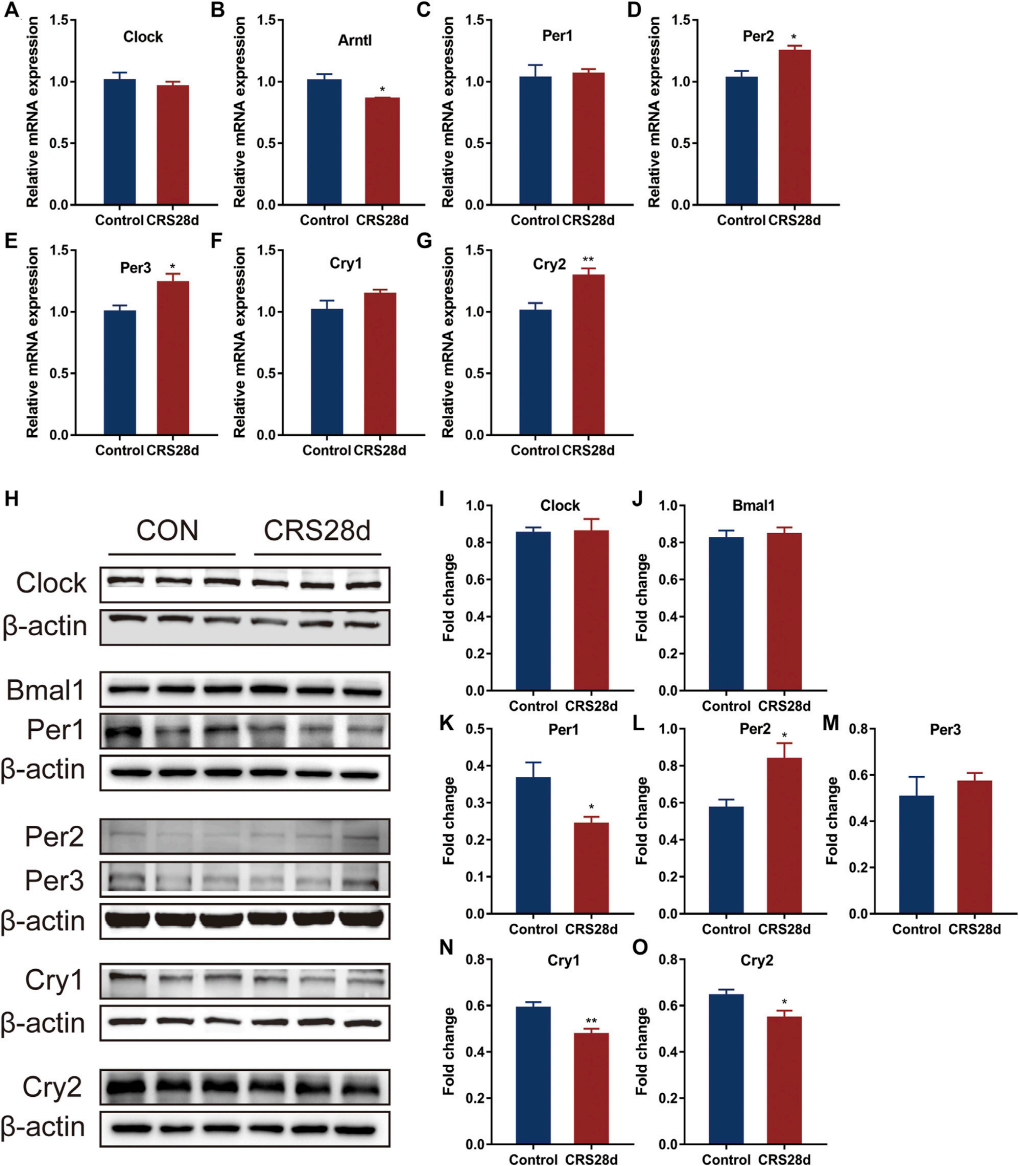
Effect of CRS 28 d on mRNA and protein expression levels of circadian rhythm genes in the mouse hypothalamus (*n* = 5–6). mRNA levels of *Clock* **(A)**, *Bmal1* **(B)**, *Per1* **(C)**, *Per2* **(D)**, *Per3* **(E)**, *Cry1* **(F)**, *Cry2* **(G)** circadian rhythm genes were examined by Real-time PCR (*n* = 5–6). **(H)** Immunoblotting of Clock, Bmal1, Per1, Per2, Per3, Cry1 and Cry2 in the hypothalamus. Quantification of Bmal1 **(I)**, Clock **(J)**, Per1 **(K)**, Per2 **(L)**, Per3 **(M)**, Cry1 **(N)**, Cry2 **(O)** protein abundance (n = 6). Data are presented as mean ± SEM, *p < 0.05, **p < 0.01.

To verify the transcriptome results, we detected the mRNA transcription of the main circadian rhythm genes forming TTFLs in the hypothalamus by qRT-PCR. The hypothalamic tissues were sampled in parallel at ZT 6.0–7.0. The relative mRNA level of *Bmal1* was significantly decreased (*p* = 0.044) while *Clock* only slightly decreased (Figures 5A,B). The results revealed a significant main effect of CRS treatment on *Per2*, *Per3*, and *Cry2* mRNA levels, which were increased in the hypothalamus (Figures 5D,E,G). The mRNA transcription of *Cry1* in CRS-treated mice was higher than in controls but not significantly so (Figure 5F). Further analysis indicated that *Per1* was unchanged (Figure 5C).

According to the results of the qRT-PCR, we then studied the protein levels of the main genes in TTFLs. Given light’s strong Zeitgeber, TTFL proteins were changed inconspicuously. There was no significant increase in Bmal1 and Clock levels in CRS-treated mice (Figures 5I,J). As described in Figure 5K–M, in response to CRS treatment, Per1–3 had different alternations. Per1 protein levels decreased significantly while those of Per2 and Per3 increased. Additional quantification analysis showed that treatment with CRS resulted in significant Cry1 and Cry2 downregulation (Figures 5N,O). These results indicated that CRS treatment influenced more the downstream factors of TTFLs, Per1–3 and Cry1–2, so we focused on their upstream regulator, melatonin.

### 3.5 CRS treatment downregulates melatonin concentration and melatonin related Trp, 5-HT and GABA

**FIGURE 6:**
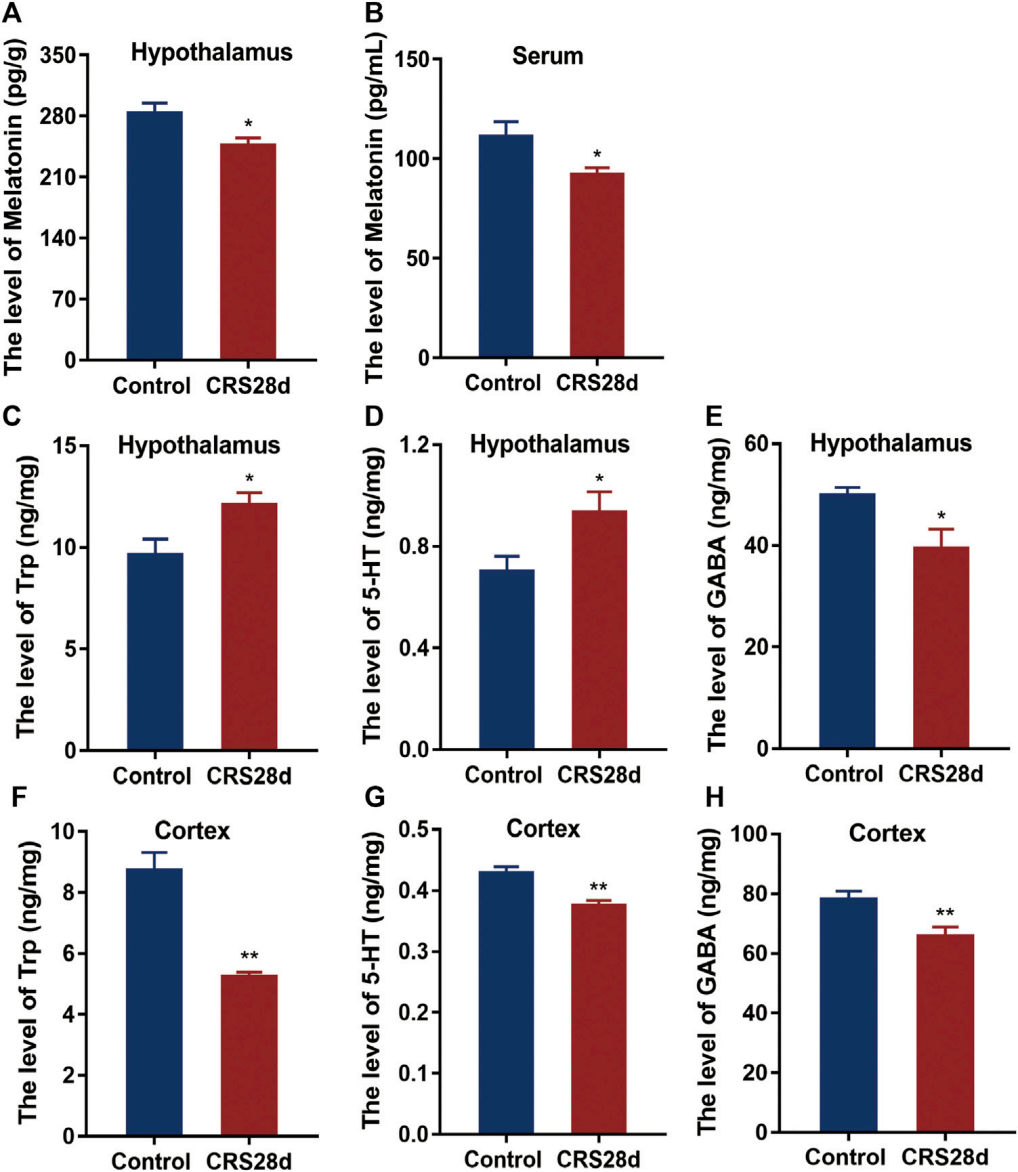
Effect of CRS 28 d on the concentrations of melatonin, 5-HT, Trp, and GABA in mice. **(A)** Melatonin in the hypothalamus (*n* = 9). **(B)** Melatonin in the serum (*n* = 10). **(C)** Trp in the hypothalamus (*n* = 10–12). **(D)** 5-HT in the hypothalamus (*n* = 8–9). **(E)** GABA in the hypothalamus (*n* = 10–11). **(F)** Trp in the cortex (*n* = 10–11). **(G)** 5-HT in the cortex (*n* = 11). **(H)** GABA in the cortex (*n* = 11–12). Data are presented as mean ± SEM, *p < 0.05, **p < 0.01.

As the circadian rhythm signaling pathway is regulated by melatonin, we measured the levels of melatonin in the brain and in the periphery. Melatonin levels were significantly lower in CRS-treated mice than in controls both in the hypothalamus and sera (Figures 6A,B). Since melatonin is synthesized from Trp and 5-HT, their concentrations in the hypothalamus and cerebral cortex were also detected. Many wake-sleep projections are sent to the cortex through different neurons to regulate sleep (Saper et al., 2010). Trp and 5-HT levels in the hypothalamus were significantly increased but dramatically decreased in the cortex (Figures 6C,D,F,G). Melatonin could increase GABA level after pinealectomy and potentiate the GABA neuronal activity (Acuña-Castroviejo et al., 1995). As described in Figures 6A,E,H significant reduction of GABA levels was observed in both the hypothalamus and cortex. These results suggest that both melatonin synthesis and maintenance were altered by CRS treatment.

### 3.6 CRS treatment decreased the transcription and expression of melatonin receptors and altered melatonin receptors’ downstream effectors

**FIGURE 7:**
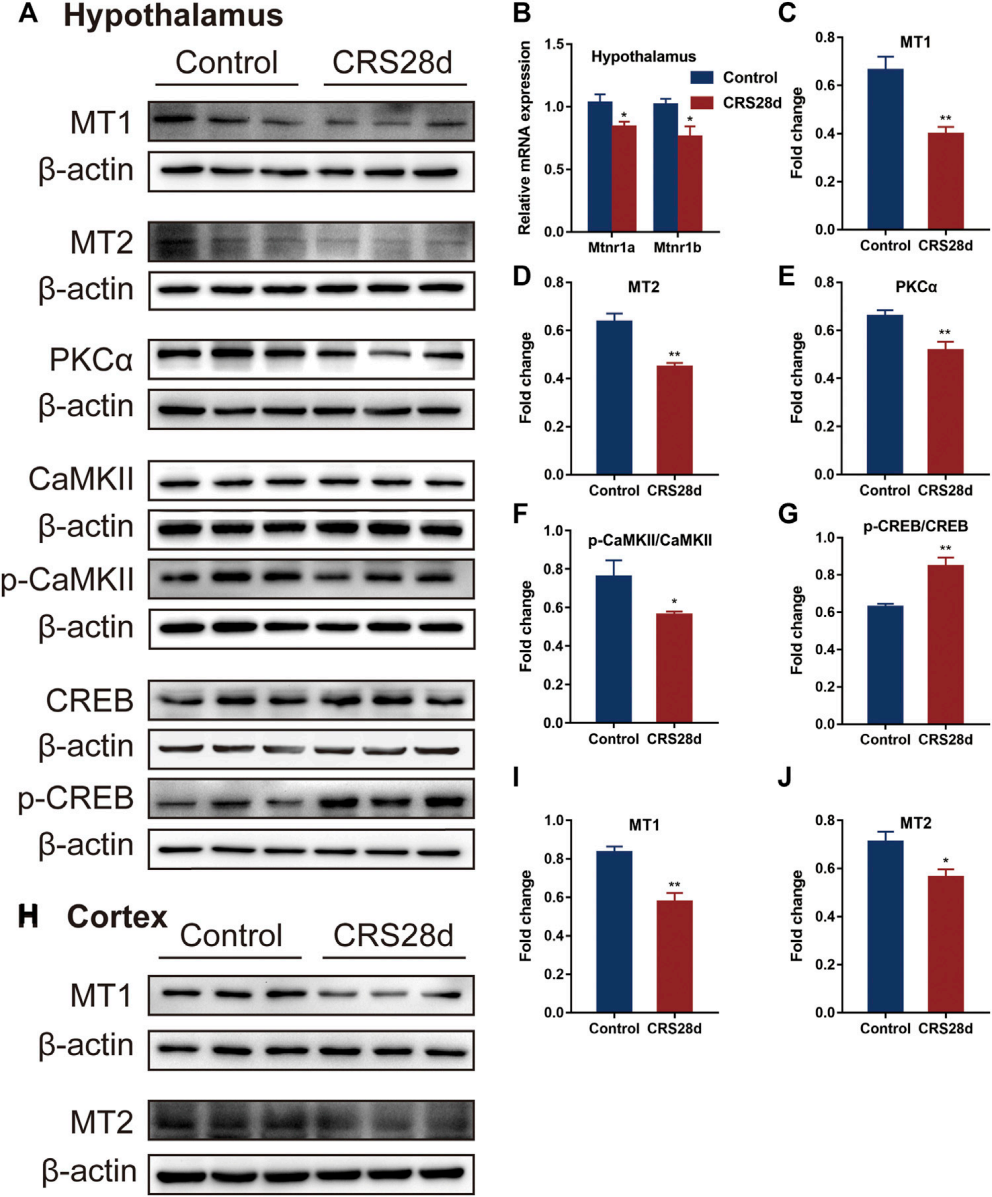
Effect of CRS 28 d on melatonin receptors and their related proteins in mice. **(A)** Immunoblotting of melatonin receptors and their related proteins in the hypothalamus. **(B)** mRNA levels of melatonin receptors (*n* = 4–6). Quantification of MT1 **(C)**, MT2 **(D)**, PKCα **(E)**, p-CAMKII/CAMKII **(F)**, p-CREB/CREB **(G)** protein abundance (*n* = 4–6) in the hypothalamus. **(H)** Immunoblotting of melatonin receptors in the cortex. Quantification of MT1 **(I)**, MT2 **(J)** protein abundance (*n* = 6) in the cortex. Data are presented as mean ± SEM, *p < 0.05, **p < 0.01.

We also investigated mRNA transcription and protein levels of the melatonin receptors MT1 and MT2 in the hypothalamus. Both were significantly downregulated by CRS treatment (Figures 7B–D). In the cortex, MT1 and MT2 protein levels were also lower compared with the control (Figures 7H–J). Further, the expression of MT downstream proteins in the hypothalamus such as protein kinase C alpha type (PKCα) and phosphorylated calcium/calmodulin-dependent protein kinase II (p-CaMKII) was significantly decreased by CRS treatment but the total CaMKII remained unaffected (Figures 7E,F). With respect to the downstream signals of MT1 and MT2, CRS treatment upregulated the phosphorylation level of cyclic adenosine monophosphate (cAMP)-responsive element binding (p-CREB) protein but CREB levels remained unchanged (Figure 7G). These data demonstrated that CRS treatment affected the MT1 and MT2 signaling pathways and that melatonin and its receptors are the major targets in the mice of sleep disorders by CRS treatment.

## 4 Discussion

**FIGURE 8:**
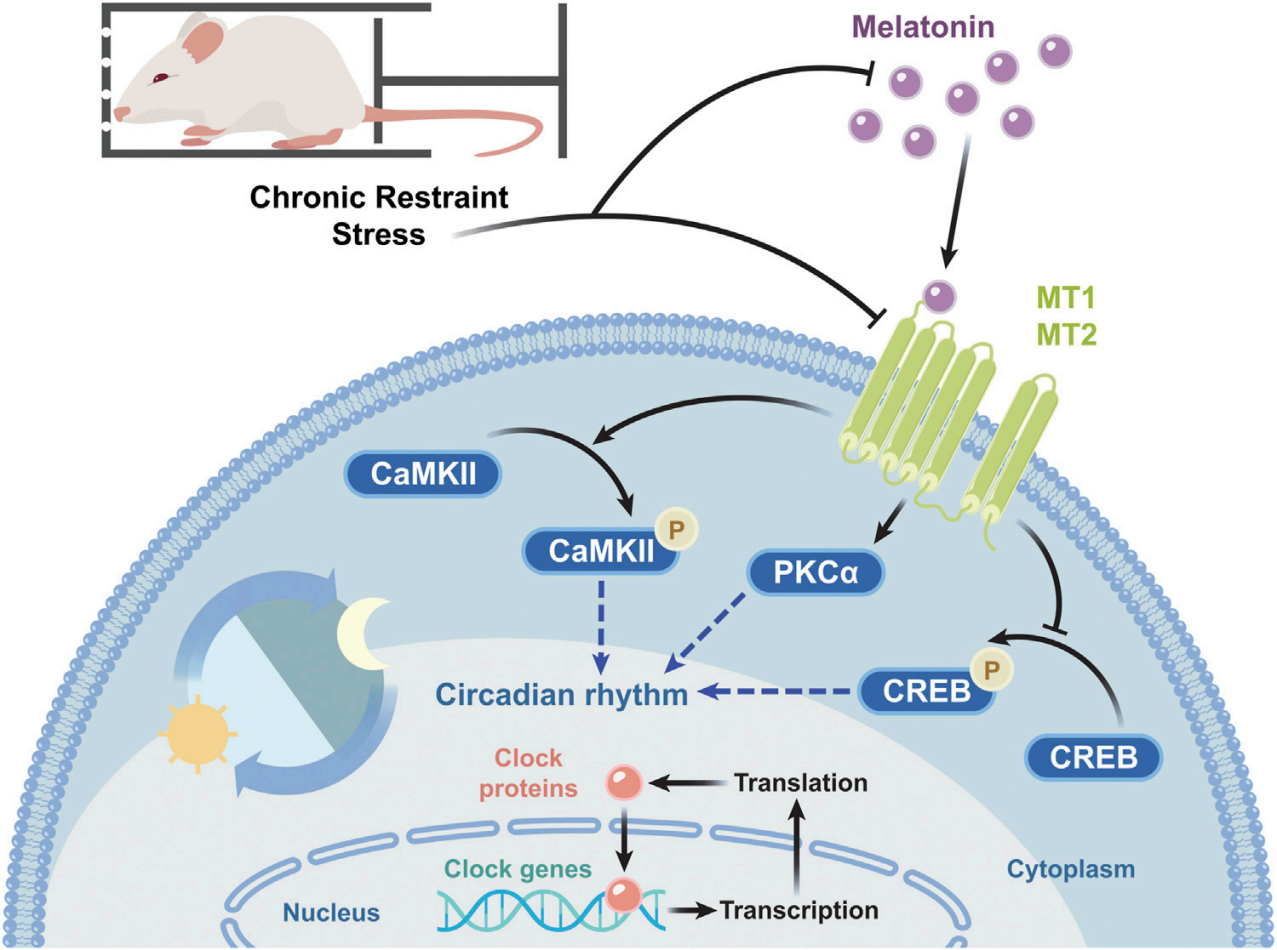
Schematic diagram of the molecular target pathways involved in CRS-induced sleep disorders. CRS decreases the melatonin level and the mRNA and protein levels of melatonin receptors, in turn reducing PKCα expression and p-CaMKII/CaMKII levels, increasing p-CREB/CREB levels, and changing the mRNA transcription and protein expression of circadian rhythm genes.

In the present study, we have simulated clinically observed chronic physiological and psychological stress in mice and explored changes in sleep architecture and molecular mechanisms underlying said sleep disorders. With the increasing CRS-modeling period, the symptoms of sleep disorders are gradually aggravated in mice. Although mice modeled by CRS for 21 days showed reduced locomotor activity and decrease in falling asleep rate and sleep duration in PST, at 28 days we observed significant sleep disorders. To our knowledge, this is the first study to examine the effects of chronic stress on mouse sleep by applying CRS for up to 28 days. Not only did the stress alter sleep architecture, but modeling for 10 h a day disrupted the mice’s circadian rhythms regulated by melatonin. Both the levels of melatonin and melatonin receptors were decreased in CRS-treated mice. The expression of melatonin-related receptors and circadian rhythm genes was affected as well. The molecular target pathways of CRS-induced sleep disorders are summarized in Figure 8.

In this study, we first found weight loss and decreased locomotor activity in mice with CRS treatment, similar to previous reports (Wang et al., 2021). The effect of CRS-treatment on sleep was evaluated by PST which preliminarily revealed reduced falling asleep rate, and shortened sleep latency and sleep duration (Table 2; Figures 1E,F). With increased CRS-treated time, sleep problems gradually intensified. Since 28 d-CRS mice showed the most obvious changes in various indexes, we chose this modeling intensity for the study of sleep architecture.

The corresponding polysomnographic of depressed patients is usually marked by a deficit in total sleep and slow wave sleep, shortened REM sleep onset latency, increased REM duration and density, sleep continuity disruption, and early morning awakening (Gillin et al., 1979; Pandi-Perumal et al., 2020). In our study, compared with the control, the CRS-treated mice had increased wakefulness in the light phase, especially during ZT 6–8 (Figure 2C). Recently, prolonged (about 4 w) chronic sleep disorders induced by psychophysiological stress have been shown to lead to increased arousal in mice (Oishi et al., 2020). The higher episode numbers of wake and NREM led to greater NREM sleep fragmentation during the light phase (Figures 3B,D). It was also confirmed by shorter average duration of NREM bouts in CRS-treated mice (Figure 3C). Correspondingly, the percentage of NREMS declined significantly during the light (ZT 2, 6–8). The alternation in these indexes indicated that CRS-treated mice had obvious symptoms of insomnia. In contrast to NREMS, the percentage, episode duration, and episode number of REMS dramatically increased (Figure 2E; Figures 3E,F). The increase in REMS was most evident in ZT14, 18–22 during the dark phase. Compared with the control mice, model mice were found to have reduced NREMS in the light phase and increased REMS in the dark phase, which demonstrated circadian rhythm disorders in CRS-treated mice (Figures 2C–E). The REMS amount and episode number also increased in the model of water immersion and restraint (2 h/d) for 1 w, 2 w, and 3 w. However, the model of water immersion and restraint showed increased wakefulness and NREMS, which is not consistent with our results because of the differences in stress factors, stimulating strength, and EEG/EMG detection period (Yasugaki et al., 2019). Another study found that 9 weeks of unpredictable chronic mild stress caused increased REMS variables in mice, which is the earliest marker of a stress response (Nollet et al., 2019). In addition, shortened REM latency has been considered a biological marker of depression in human (Palagini et al., 2013). In our study, the REMS latency was reduced totally in CRS-treated mice but more significantly during the dark phase (Figure 3G). The REMS latency was also decreased in the mice under social defeat stress for 10 days (Wells et al., 2017). In summary, CRS treatment caused sleep fragmentation, circadian rhythm disorders, and insomnia in mice is consistent with clinical observations from depressive patients and demonstrated typical stress-associated sleep disorders (Crouse et al., 2021).

Melatonin inputs to sleep controlling neurons reduce the time to sleep onset and increase sleep duration, also increasing NREMS (Holmes and Sugden, 1982). Research demonstrated that melatonin levels were significantly reduced at the peak of secretion in depression patients (Ogłodek et al., 2016). In our results, melatonin levels were reduced not only in the hypothalamus but also in the serum, indicating an overall decrease in central and peripheral melatonin levels. Trp and 5-HT levels were increased in the hypothalamus but decreased in the cortex, suggesting an issue with melatonin synthesis, which may be related to the activity of aralkylamine-N-acetyltransferase and N-acetylserotonin-O-methyltransferase enzymes. Studies over the past three decades has shown that 5-HT functions predominantly to promote wake (Monti, 2011). Increased hypothalamic 5-HT levels explained the increased wakefulness in model mice. Cortical 5-HT decline has been reported in CRS model (Geng et al., 2021; Sahoo et al., 2021), but its association with sleep needs further investigation. Melatonin increases GABA concentration by stimulating glutamic acid decarboxylase (Rosenstein and Cardinali, 1990), the enzyme that synthesizes GABA from glutamic acid. The ventrolateral preoptic nuclei is the main sleep-promoting center and contains plenty of GABAergic neurons, which inhibit the major wake-promoting centers in the hypothalamus and brain stem by GABA to induce sleep (Riemann et al., 2020). In our study, the significant decrease in GABA levels in the hypothalamus and cortex, which affect sleep in CRS-treated mice, may be related to the decreased melatonin levels in the brain.

The mRNA transcription of *MT1* and *MT2* is not only decreased in the hypothalamus, but the protein expressions are also decreased in the hypothalamus and cortex. In mice with genetic inactivation of both MT1/MT2 (*MT1*−/−/*MT2*−/−) receptors, wakefulness time increased. Mice with single inactivation of MT1 receptors showed a decrease in REMS time whereas those with inactivated MT2 receptors displayed a decrease in NREMS time (Comai et al., 2013). Therefore, MT1 receptors are mainly involved in REM sleep regulation while MT2 receptors are in NREM sleep (Gobbi and Comai, 2019). In our study, aside from the lower NREMS time in CRS-treated mice, we found significantly lower levels of both MT1 and MT2 receptors. Between *MT1*−/−/*MT2*−/− and wild-type mice, the total time of NREMS showed a downward trend but no statistical difference (Comai et al., 2013). Therefore, our study found a tendency that CRS treatment affected not only the expression of MT1 and MT2 receptors but other factors regulating sleep.

Downstream effectors of MT receptors play important roles in circadian rhythms and sleep regulation. In fact, melatonin receptors are coupled to pertussis toxin-sensitive G proteins. Pertussis toxin blocked melatonin-induced phase shifts in MT1 receptor-deficient mice (Liu et al., 1997). Melatonin can selectively activate PKC in the SCN inducing phase shift. For instance, 12-O-Tetradecanoylphorbol 13-acetate, a specific and direct PKC activator, could mimic the effect of melatonin, while a PKC inhibitor blocked this effect (McArthur et al., 1997). In our study, the reduction of PKCα proteins in the hypothalamus was decreased. Furthermore, the activation of MT2 receptors increases intracellular calcium ion and CaMKII expression and reduces CREB phosphorylation to recover the delayed sleep phase (Wang et al., 2020). CRS-treated mice showed decreased p-CaMKII levels and increased p-CREB levels. These results indicated impaired melatonin receptor function and downstream signal transduction that eventually affects the circadian rhythm.

Melatonin, melatonin receptors, and downstream proteins affect mRNA transcription and protein expression of circadian rhythm genes. Dardente et al. demonstrated that *Cry1* mRNA transcription (with diminished Per1) was directly induced by melatonin in the pars tuberalis (Dardente et al., 2003). Melatonin affects classical receptor-mediated pathways, regulating the circadian rhythm proteins via phosphorylation (Von Gall et al., 2002) and proteasome pathway (Vriend and Reiter, 2015). In addition, PKCα overexpression suppresses Bmal1-Clock transcriptional activity (Robles et al., 2010). The expression of circadian factors Bmal1, Clock, Per, and Cry is mainly regulated by the CaMK-CREB signaling pathway in the SCN (Yokota et al., 2001). Melatonin pretreatment can restore the circadian process by regulating circadian factors expressions through the CaMK-CREB pathway and post-translational modulation (Yin et al., 2022). With respect to mRNA, the transcription of *Per1–3* and *Cry1–2* was increased. The protein levels of Per1, Cry1, and Cry2 significantly decreased in the hypothalamus whereas those of Per2 increased after CRS treatment. Another research showed the relationship between melatonin and the rhythmic pattern of clock genes and their proteins in the pars tuberalis (Jilg et al., 2005). The mRNA transcriptions and protein expressions of clock genes have their own rhythm, and there is no clear expression correspondence. The changes in circadian rhythm genes between the CRS-treated and control mice suggested that CRS treatment induced circadian rhythm disorders, related to sleep disorders.

In conclusion, our results indicate that chronic CRS treatment induces typical stress-associated sleep disorders in mice which is consistent with observations from patients. All observed sleep disorders are related to impaired melatonin pathways and manifested by decreased levels of ligands, receptors, and relative downstream effectors. This study accessed the cause of stress-associated sleep symptoms. This study will also help us better understand the sleep characteristics and the pathogenesis of insomnia in depressed patients and provide a firm basis for future research. However, the changes in melatonin-related pathways may not fully explain the cause of CRS-induced sleep disorders; in the future, more targets need to be explored to dissect the complete mechanisms.

## Data Availability

The data presented in the study are deposited in the Figshare Dryad Digital Repository, and can be found online at: https://figshare.com/s/e19f30e8328125580863. Further inquiries can be directed to the corresponding authors.
