# Supplementary material for: Melatonin-related dysfunction in chronic restraint stress triggers sleep disorders in mice
Source: Front Pharmacol. 2023 Jun 20;14:1210393. doi: 10.3389/fphar.2023.1210393 (PMC10318904; doi:10.3389/fphar.2023.1210393)

Supplementary Material

Supplementary Figure 1. Original image files of western blots in **figure 4**. For each protein, six samples in the group were assigned to two bolts. For each blot, the first three samples were from the control group, while the last three samples were from the CRS 28d group.

1. Clock

Clock-a

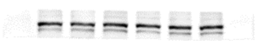

Internal reference protein (β-actin) for Clock-a

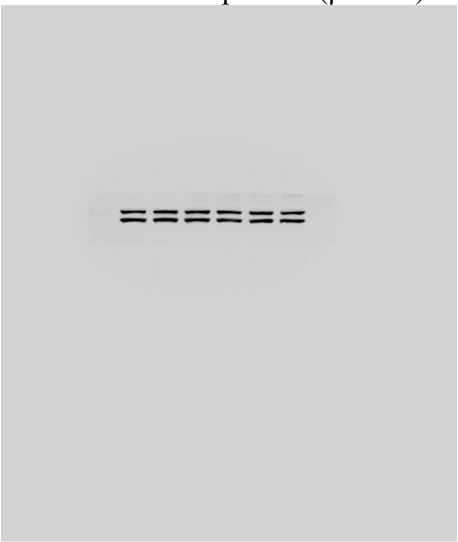

Clock-b

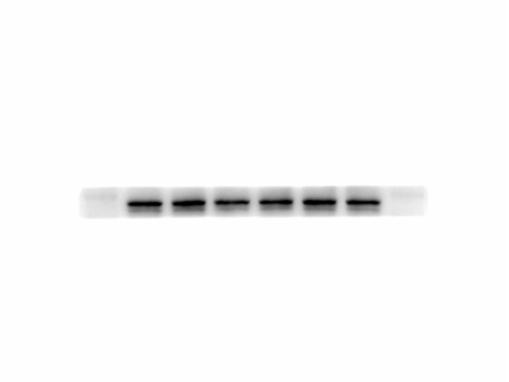

Internal reference protein (β-actin) for Clock-b

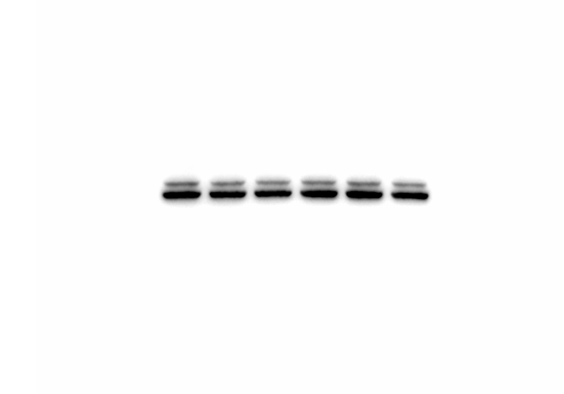

2. Bmal1

Bmal1-a

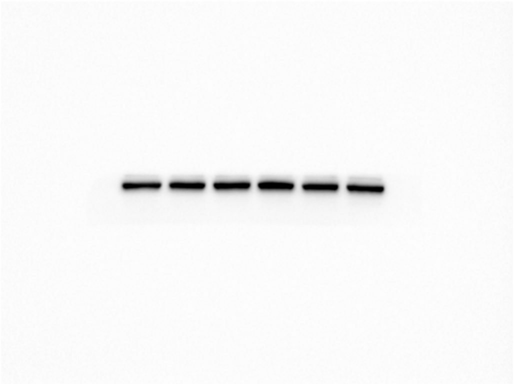

Internal reference protein (β-actin) for Bmal1-a and Per1-a

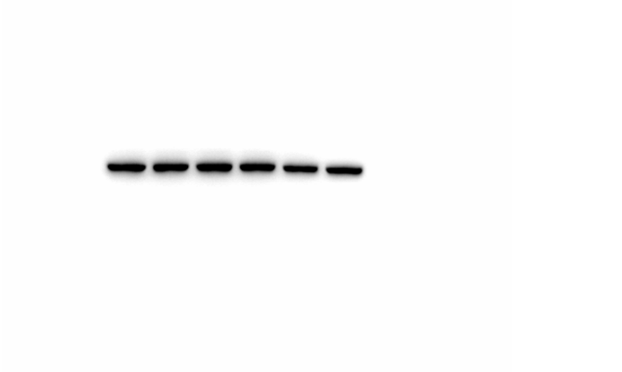

Bmal1-b

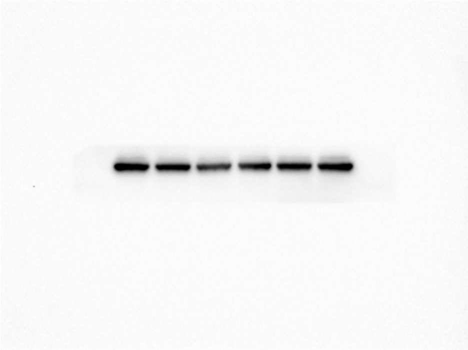

Internal reference protein (β-actin) for Bmal1-b

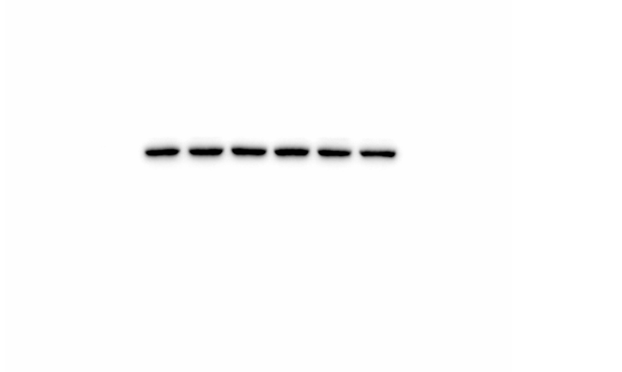

3. Per1

Per1-a

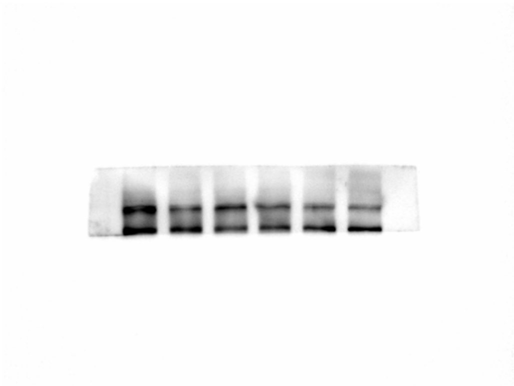

Internal reference protein (β-actin) for Per1-a and Bmal1-a

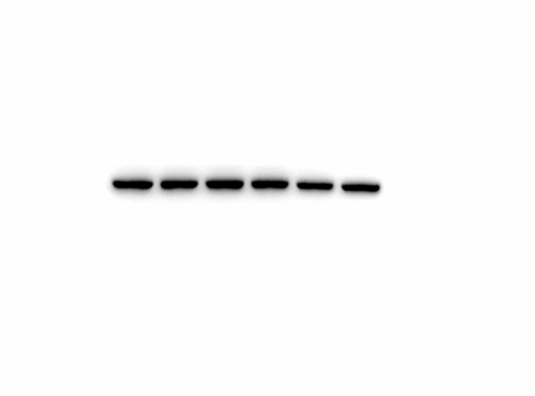

Per1-b

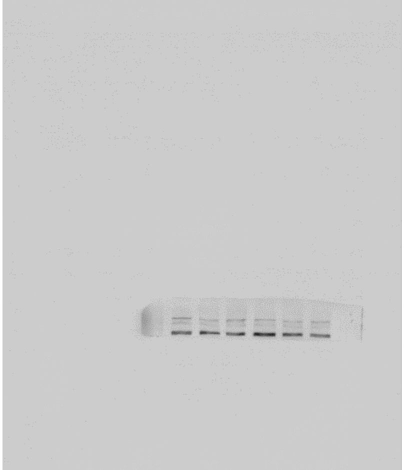

Internal reference protein (β-actin) for Per1-b

4. Per2

Per2-a

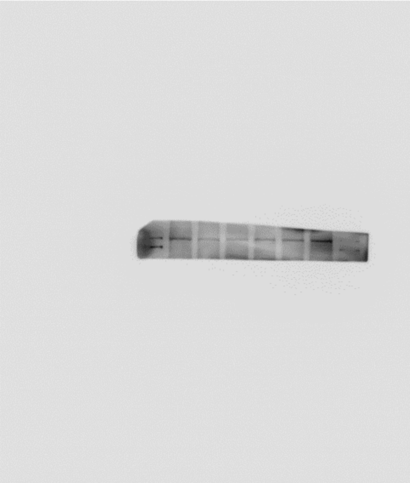

Internal reference protein (β-actin) for Per2-a and Per3-a

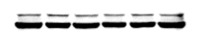

Per2-b

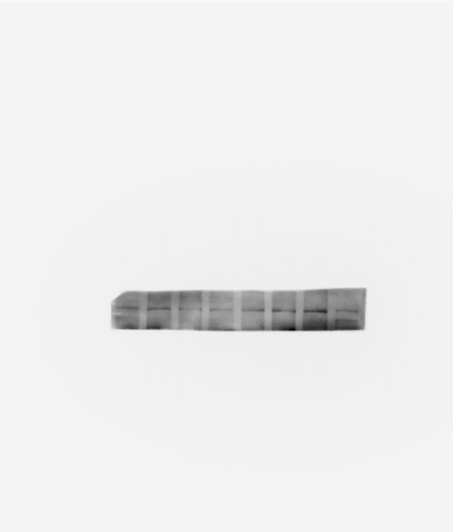

Internal reference protein (β-actin) for Per2-b

5. Per3

Per3-a

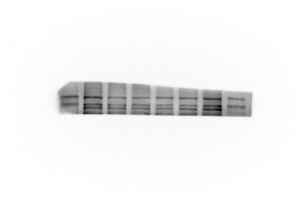

Internal reference protein (β-actin) for Per3-a and Per2-a

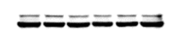

Per3-b

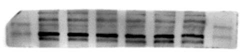

Internal reference protein (β-actin) for Per3-b and Cry1-b

6. Cry1

Cry1-a

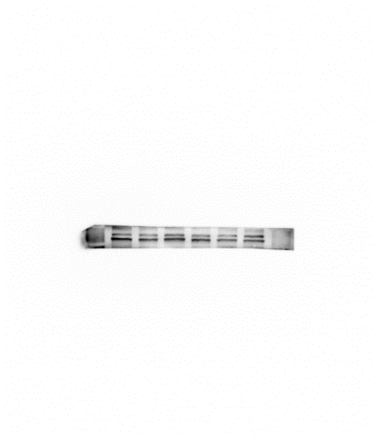

Internal reference protein (β-actin) for Cry1-a

Cry1-b

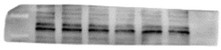

Internal reference protein (β-actin) for Cry1-b and Per3-b

7. Cry2

Cry2-a

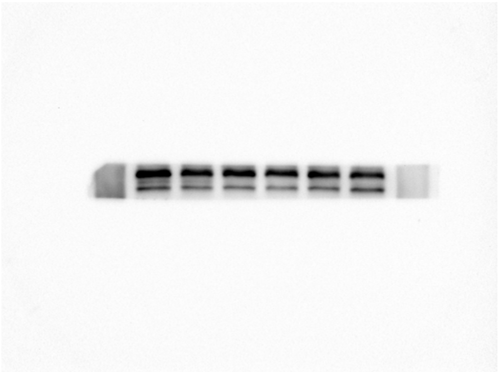

Internal reference protein (β-actin) for Cry2-a

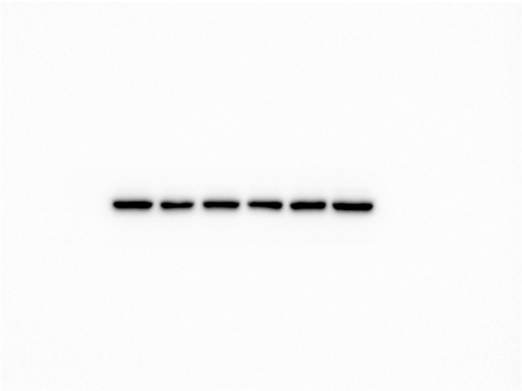

Cry2-b

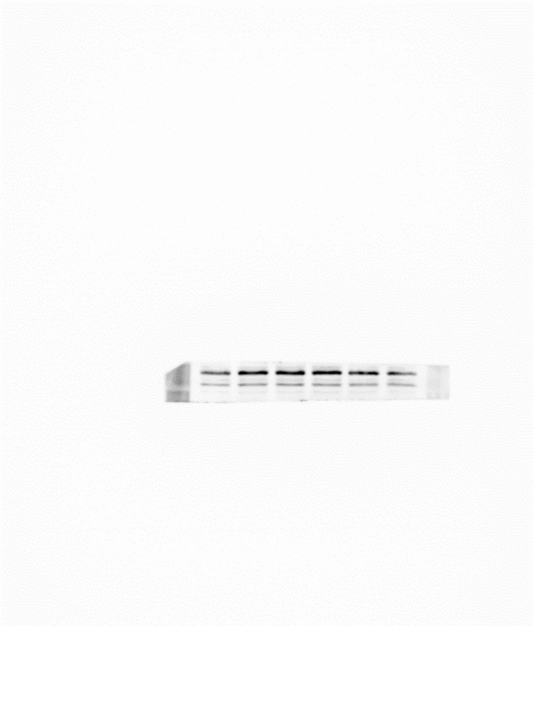

Internal reference protein (β-actin) for Cry2-b

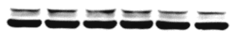

Supplementary Figure 2. Original image files of western blots in **figure 5**. For each protein, six samples in the group were assigned to two bolts. For each blot, the first three samples were from the control group, while the last three samples were from the CRS 28d group.

Hypothalamus

1. MT1

MT1-a

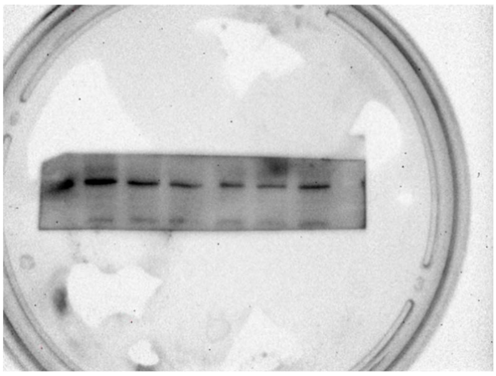

Internal reference protein (β-actin) for MT1-a

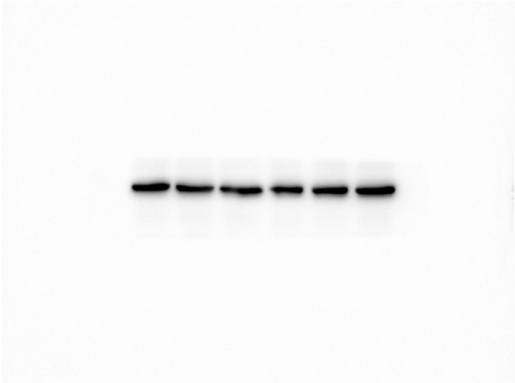

MT1-b

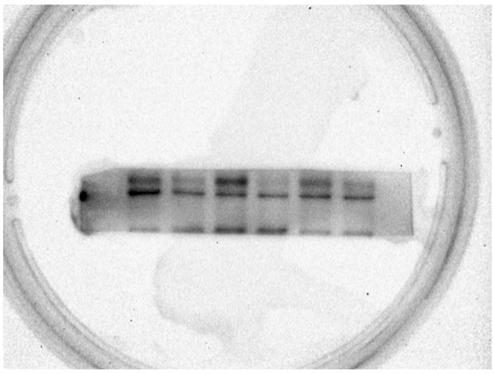

Internal reference protein (β-actin) for MT1-b and p-CREB-a

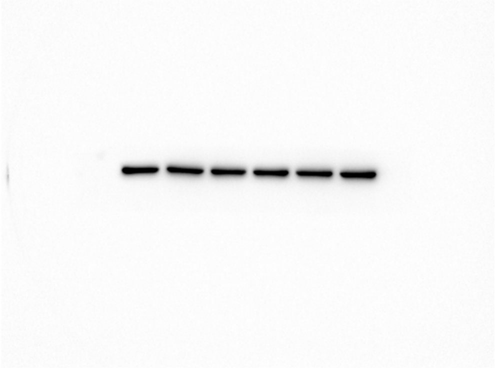

2. MT2

MT2-a

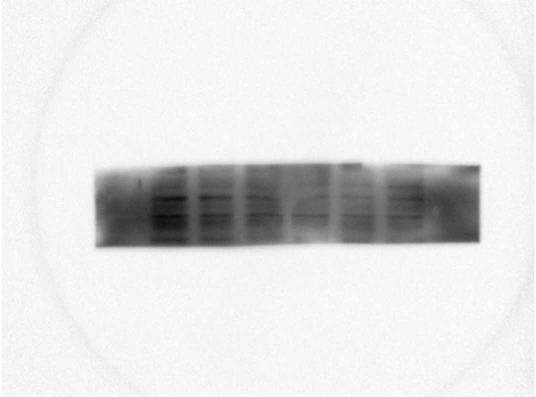

Internal reference protein (β-actin) for MT2-a

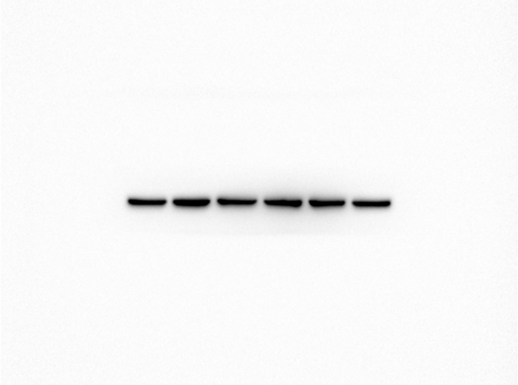

MT2-b

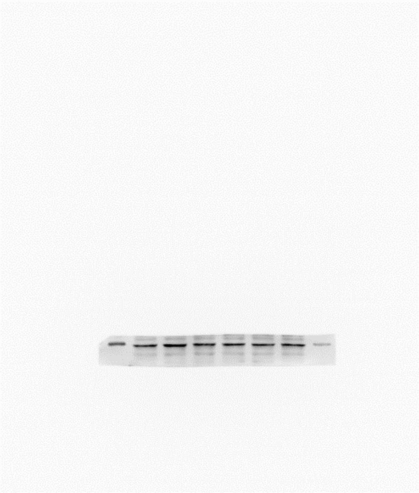

Internal reference protein (β-actin) for MT2-b

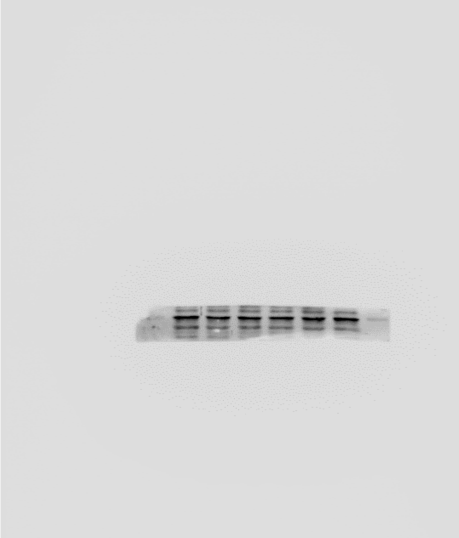

3. PKCα

PKCα-a

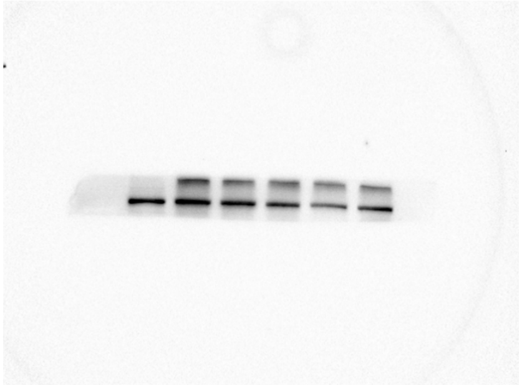

Internal reference protein (β-actin) for PKCα-a

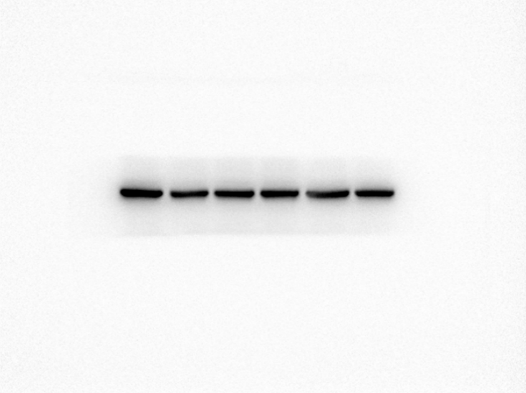

PKCα-b

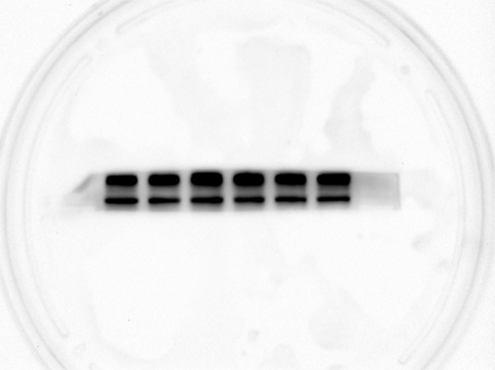

Internal reference protein (β-actin) for PKCα-b

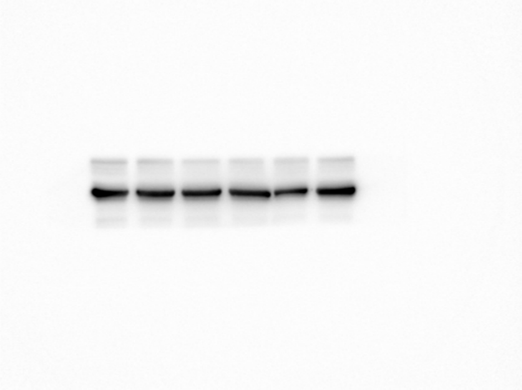

4. CaMKII

CaMKII-a

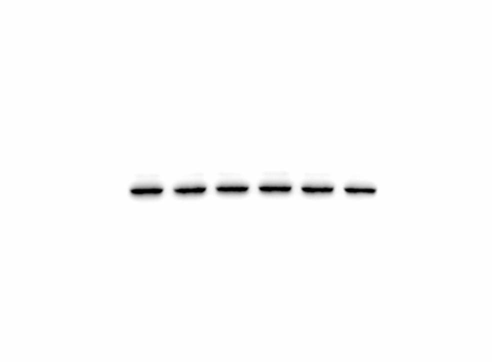

Internal reference protein (β-actin) for CaMKII-a

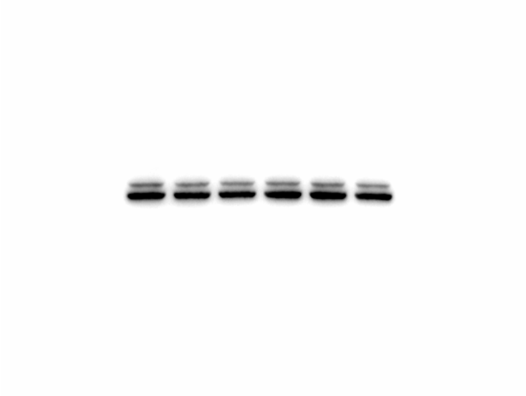

CaMKII-b

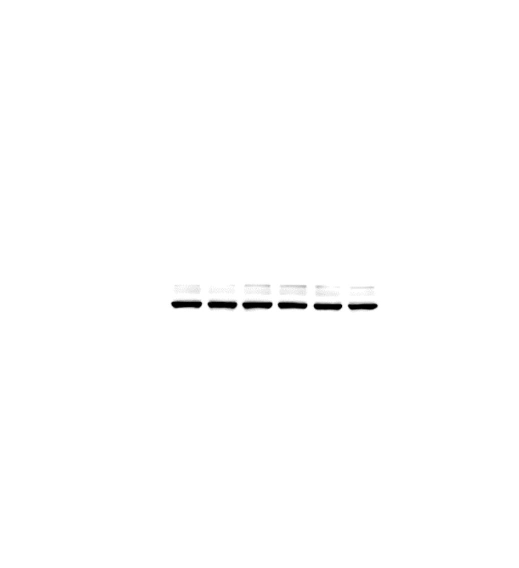

Internal reference protein (β-actin) for CaMKII-b

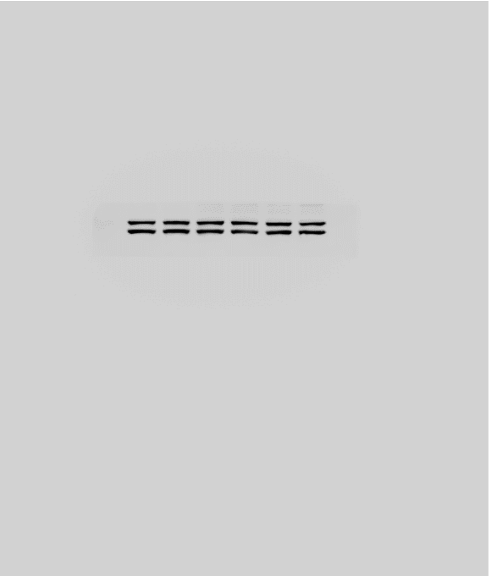

5. p-CaMKII

p-CaMKII-a

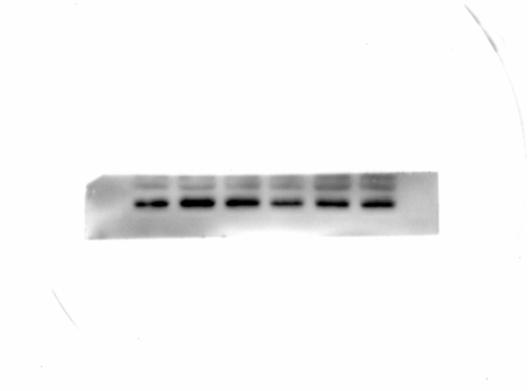

Internal reference protein (β-actin) for p-CaMKII-a

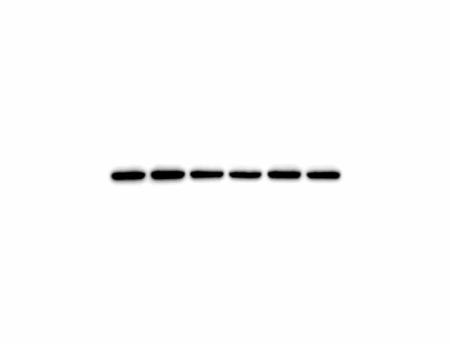

p-CaMKII-b

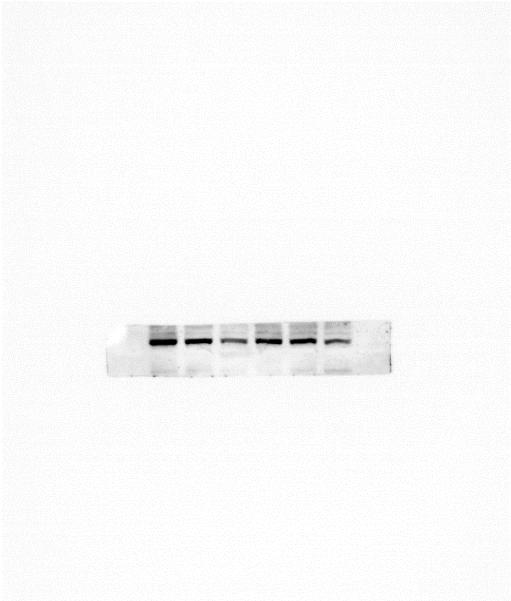

Internal reference protein (β-actin) for p-CaMKII-b

6. CREB

CREB-a

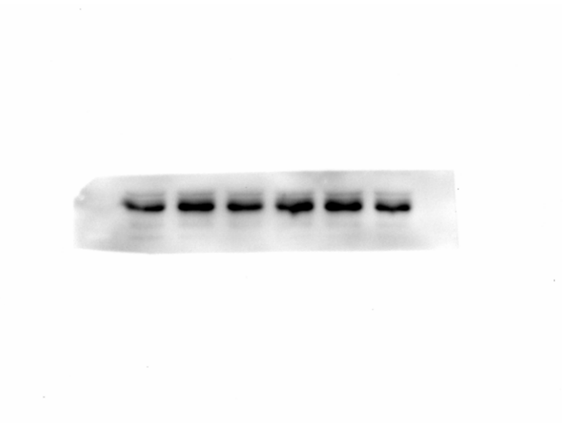

Internal reference protein (β-actin) for CREB-a

CREB-b

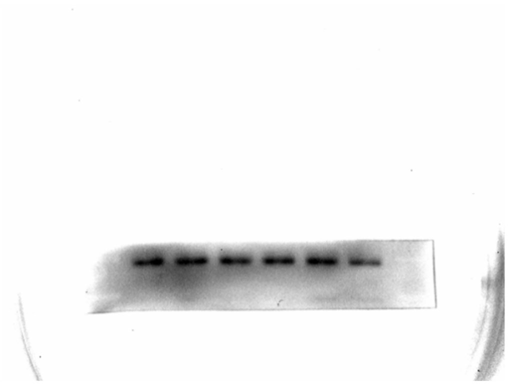

Internal reference protein (β-actin) for CREB-b

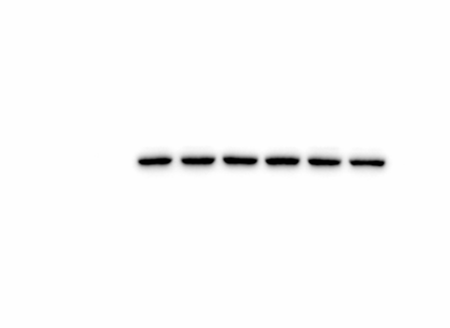

7. p-CREB

p-CREB-a

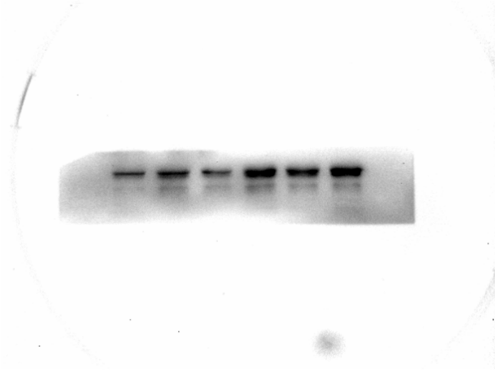

Internal reference protein (β-actin) for p-CREB-a and MT1-b

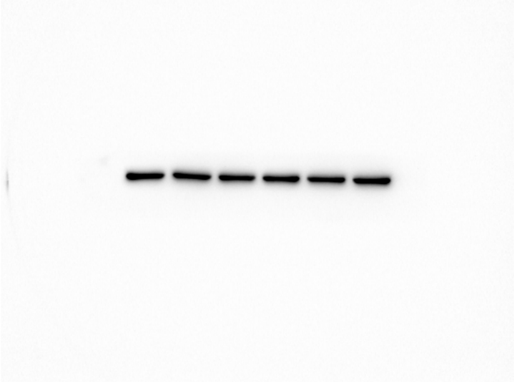

p-CREB-b

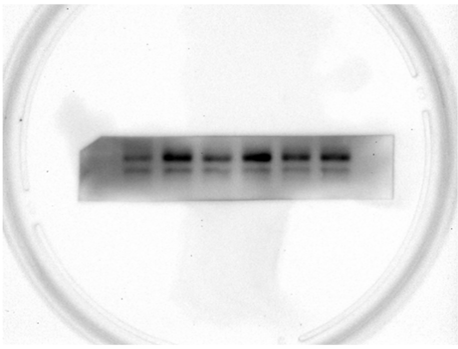

Internal reference protein (β-actin) for p-CREB-b

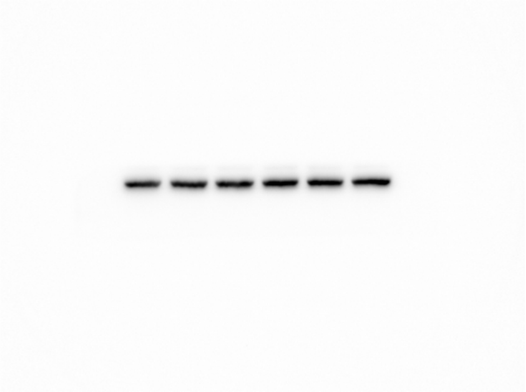

Cortex

1. MT1

MT1-a

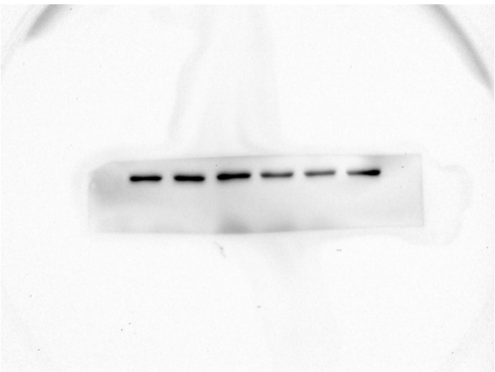

Internal reference protein (β-actin) for MT1-a

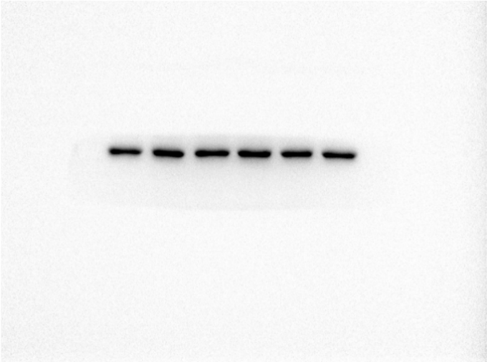

MT1-b

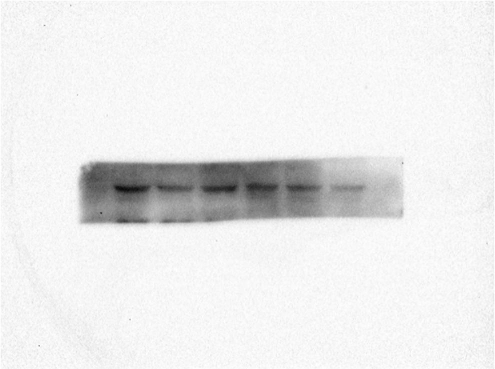

Internal reference protein (β-actin) for MT1-b

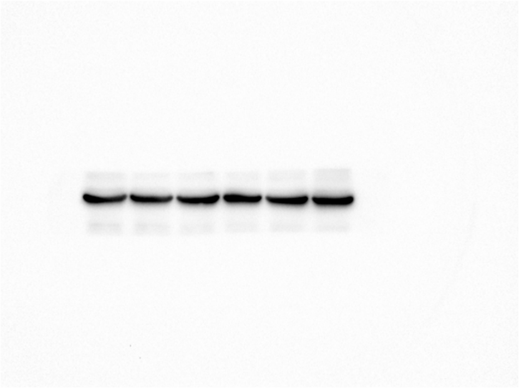

2. MT2

MT2-a

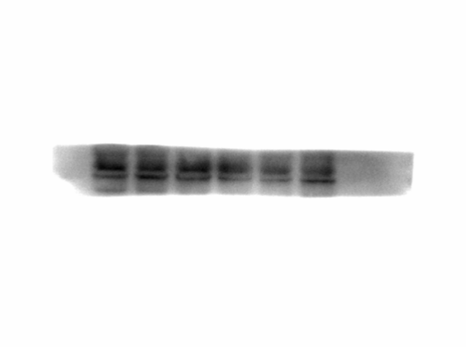

Internal reference protein (β-actin) for MT2-a

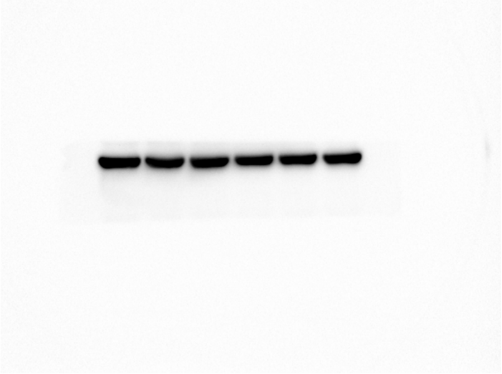

MT2-b

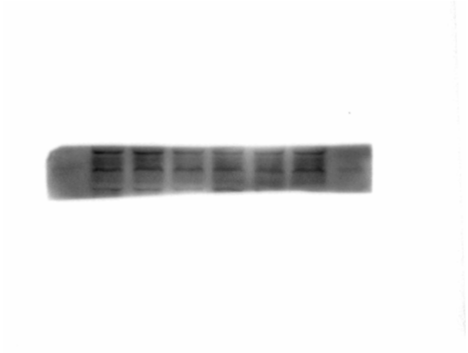

Internal reference protein (β-actin) for MT2-b

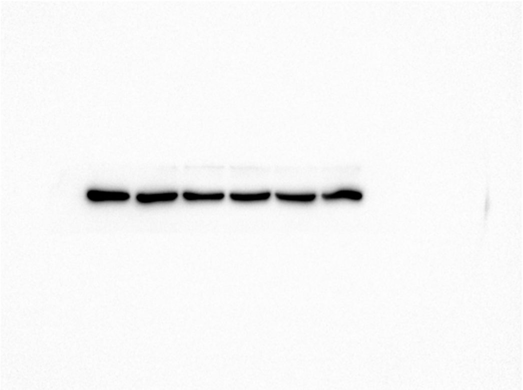

Supplement: Supplementary file 1 [file DataSheet1.PDF]
